# Validation of a novel method of ultraviolet-induced cutaneous inflammation and its associations with anhedonia

**DOI:** 10.1038/s41598-022-24598-4

**Published:** 2022-11-24

**Authors:** Holly Sullivan-Toole, Shengchuang Feng, Corinne N. Carlton, Merage Ghane, Thomas M. Olino, Irving C. Allen, John A. Richey

**Affiliations:** 1 Department of Psychology, Virginia Tech, Blacksburg, USA; 2 Fralin Biomedical Research Institute at VTC, Roanoke, USA; 3 Department of Psychiatry, University of Pittsburg, Pittsburg, USA; 4 Department of Psychology and Neuroscience, Temple University, Weiss Hall, 1701 N 13Th St, Philadelphia, PA 19122 USA; 5 Department of Biomedical Sciences and Pathobiology, Blacksburg, VT USA

**Keywords:** Acute inflammation, Human behaviour, Skin manifestations

## Abstract

Affective immunology of the skin is a growing area; however, established protocols for measuring individual differences in cutaneous inflammation are lacking. To address this, we present a preliminary validation of Precision Implementation of Minimal Erythema Dose (PI-MED) testing as a method for measuring cutaneous inflammation. PI-MED is a recently adapted protocol, optimized for reproducibility and individual differences research, that uses ultraviolet (UV) light to evoke cutaneous erythema, or inflammatory skin reddening. PI-MED’s novel UV dosage schedule produces standardized erythema responses across different skin pigmentation types and shows strong internal consistency within person and good test–retest reliability across 8–10 weeks. In line with predictions, increased PI-MED erythema was associated with heightened anhedonia, across several measures, beyond influences of non-affective covariates. While future work should further refine the dosage schedule for the lightest and darkest skin types, overall, evidence supports PI-MED as a protocol for inducing and measuring individual differences in cutaneous inflammation. Further, PI-MED-induced erythema can expand psychoneuroimmunology research by offering a complementary assessment for general inflammatory tone. This work adds to a growing body of evidence demonstrating a distinct relationship between inflammation and anhedonia.

## Introduction

Affective immunology of the skin is a growing area of research^1,2^. The affective and immune systems engage in co-regulation of one another^3,4^, and the skin has been referred to as a ﻿‘neuroimmunoendocrine interface’, serving as a central site for interactions between the nervous, immune, cutaneous, and endocrine systems^5–11^. Inflammation, a process of coordinated delivery of blood components to defend against pathogens, is an important mechanism through which affective processes and health influence one another, and inflammation is implicated in multiple psychiatric conditions^4,12,13^. Affective processes also interact with skin specifically^1,7^, and inflammation serves an important function in this neuro‐immuno‐cutaneous communication^6,14^. While substantial work has examined relationships between affect and inflammation markers sampled from peripheral fluids^15–18^, surprisingly little is known about affect and cutaneous inflammation.

While established protocols and best practices exist for measuring inflammatory parameters from blood and saliva [e.g.,^19–22^], protocols for measuring inflammation in skin are not widely established. Yet, there is a need for improved measurement and/or additional approaches to understand psycho-neuro-inflammatory processes in humans. For example, inflammation markers often show inconsistent or weak associations across blood-based and salivary measures^19,22^, suggesting additional measurement modalities may help elucidate complex inflammatory processes. The investigation of cutaneous inflammation is a relatively novel and promising avenue for psychoneuroimmunology research that could expand knowledge about inflammatory processes in health even beyond the skin. Indeed, immune dysfunction in epithelial tissue (e.g., skin, gut) is thought to play an important precipitating role in broader systemic inflammation^23,24^. However, little is known about how cutaneous inflammation is associated with systemic inflammation and/or inflammatory parameters from peripheral fluids. While the emerging field of psychoneurocutaneous medicine considers affect and skin intimately connected^1,2^, there is a lack of research jointly investigating affect and inflammatory processes in relation to human skin.

Precision Implementation of Minimal Erythema Dose (PI-MED) testing^25^, an adaptation of the Minimal Erythema Dose (MED) procedure^26^, is a minimally invasive procedure for the precise measurement of inflammatory response to ultraviolet (UV) light exposure, optimized for investigating individual differences in cutaneous erythema (inflammatory reddening of skin). UV-induced inflammation is highly relevant to biological mechanisms of interest in psychoneuroimmunology and inflammation-related research generally^9^. Pro-inflammatory cytokines IL-6, ﻿TNF-α, and IL-1β, commonly associated with depression^4,17,27,28^ and implicated in the neuroinflammation linked to psychiatric symptoms^12^, are known to be stimulated in the skin and blood by UV exposure. For example, TNF-α, IL-10, and IL-1β increase in UV-irradiated human skin in a time- and dose-dependent manner^29^. Further, UV exposure stimulates secretion of IL-6, ﻿TNF-α, and IL-1β in keratinocytes within irradiated skin^30,31^, which then further stimulate the release of pro-inflammatory cytokines from non-irradiated cells including cells in peripheral blood^30^. Indeed, following UV exposure, higher levels of IL-6, ﻿TNF-α, IL-1β, and C-reactive protein are found in both skin and blood^32,33^. In addition to UV inducing local and systemic inflammatory processes, PI-MED testing may serve as an indirect assay of systemic inflammation levels. For example, a number of studies have demonstrated that consuming certain nutritional supplements with antioxidant properties, thought to ameliorate systemic inflammation, also reduces erythema response to UV^34–37^ and decreases UV-induced pro-inflammatory cytokines in the skin and blood^32^, suggesting that the body’s general inflammatory environment can be indirectly assessed via UV-induced erythema response. Thus, PI-MED offers a relatively inexpensive procedure for assessment (especially repeated assessment) of peripheral inflammatory tone compared to blood-based and salivary protocols.

Previous research using MED has demonstrated that UV-induced erythema response is strongly associated with skin type (which aligns with melanin-associated pigmentation) as well as other factors beyond skin type^38^; however, these other factors are not well characterized, and, unlike other inflammatory processes, very little is known about relationships between cutaneous inflammation and core dimensions of affect. Extensive evidence highlights interactions between affect and peripheral inflammation. Inflammatory markers extracted from peripheral fluids show associations with reduced positive and increased negative emotion^15–17,39,40^, stress^16,18,41–43^, and depression^4,17,28^.

In particular, converging research points to a strong relationship between inflammatory activation and anhedonia-related symptoms or positive valence system deactivation^17,44–50^. First, associations between inflammatory markers and anhedonia symptoms are distinct from associations with negative affect^48,51–53^, depression^40,54^, or history of recurrent depression^55^. For example, metabolite ratios consistent with activation of the ﻿kynurenine pathway, and thus indicative of a pro-inflammatory environment, were associated with anhedonia, controlling for other depression symptoms, in adolescents^56^ and with anhedonia, but not depression symptoms, in adults^57^. In rats, stress-induced anhedonia (reduced sucrose preference), rather than stress itself, was associated with increased levels of IL-6 and IL-1β^47^. Relatedly, considerable evidence in humans suggests that positive affect buffers against the known inflammatory effects of stress^58^. Additionally, experimental work in humans and animals provides more direct evidence for the strong link between diminished positive valence system activation and increased inflammation/weakened immune system functioning. In mice, experimental manipulations thought to engage the positive valence system improved immune system resiliency and inflammation resolution^3^. Similarly, experimental work in humans has demonstrated that positive mood induction enhances immune function [^59,60^, for review see^61^]. Reciprocally, in humans and animals, experimentally- or medically-induced inflammation leads to reduced responsivity to rewards and heightened anhedonia-related symptoms^61–63^. Thus, we anticipated that PI-MED-induced cutaneous inflammation would show a specific and pronounced association with anhedonia. Further, given research suggesting that social anhedonia may be dissociable from anhedonia within other reward domains^64–66^ and evidence suggesting nuanced associations between social hedonic processes and inflammation^67,68^, we were also interested in examining potentially distinct associations between erythema and social anhedonia.

Here, we present a preliminary validation of PI-MED testing^25^ as a method for precisely inducing and measuring cutaneous inflammation and present evidence for PI-MED’s relevance to psychoneuroimmunology research. Our first aim examined evidence for the precision of the PI-MED procedure. Specifically, we examined evidence for the standardization of the PI-MED erythema response across different skin pigmentation types as well as evidence for the internal reliability and test–retest reliability of the PI-MED erythema measure. Our second aim explored PI-MED’s relevance for psychoneuroimmunology research by examining associations between PI-MED-induced erythema and covariates previously associated with other measures of peripheral inflammation. The PI-MED data was collected as part of a clinical trial in which anhedonia was the major outcome of interest^69^, and given substantial evidence supporting a pronounced relationship between inflammation and anhedonia, we hypothesized that baseline anhedonia measures would show associations with PI-MED erythema, beyond effects of non-affective covariates.

## Method

### Participants and study overview

Participants provided written informed consent in accordance with the Declaration of Helsinki. Participants were drawn from a randomized controlled trial examining effects of an 8-week Mindfulness Based Stress Reduction (MBSR) intervention (compared to a waitlist control condition) in adults aged 18 to 60 reporting chronic psychological stress. Participants were recruited from a university campus and surrounding community in a rural region of the southern United States. A full description of the clinical trial and affective outcomes have been reported elsewhere^69^. The current manuscript reports only the methods and results pertinent to (1) the standardization of the PI-MED erythema response across skin pigmentation types and reliability of the PI-MED erythema measure and (2) associations between PI-MED erythema and covariates. Exclusion criteria for the clinical trial were: extensive previous meditation experience, current daily practice with mind–body techniques such as yoga, currently working a night shift, current smoker, self-reported problem with drugs or alcohol, use of steroid or corticosteroid medication, and any of the following medical conditions: hypertension, hyperlipidemia, high cholesterol, diabetes (type I or II), or a family history of coronary or atherosclerotic disease. Additionally, individuals taking psychotropic medications were excluded if they reported any changes to their medication in the previous three months, and any participants already taking psychotropic medication were discontinued in the study if they altered their medication during the study. We asked participants to refrain from using drugs that may impact immune responses in the 24 h prior to the PI-MED procedure, including: glucocorticoids, estrogen, and non-steroidal anti-inflammatory drugs (e.g., aspirin, ibuprofen, naproxen). Further, participants were asked not to use any sunscreen or lotions containing sunscreen 24 h before the PI-MED procedure. While recruitment materials targeted stressed adults, there were no inclusion/exclusion criteria related to stress levels or any psychiatric diagnoses.

To assess erythema response, PI-MED testing was conducted at baseline (prior to randomization into either the mindfulness intervention or waitlist condition) and at study end-point (approximately 8—10 weeks after baseline assessments and after completion of the intervention/waitlist period). Potential covariates of inflammation were collected at baseline including non-affective covariates (e.g., age, body mass index) and self-report measures of affect. At baseline, sixty-one participants completed PI-MED. At study end-point, PI-MED data were not collected from 18 participants (end-point n = 43) for the following reasons: seven participants withdrew from the study; five participants had scheduling conflicts that interfered with end-point data collection; and, out of an abundance of caution, the PI-MED procedure was not conducted on six participants due to residual discoloration from PI-MED implemented at baseline (approximately 8–10 weeks prior), or because the participant opted out. See Table 1 for participant demographics.

**Table 1:** Summary statistics for sample demographics and other variables of interest.

Baseline participants (n = 61)	Mean (SD) or total	Percentage of sample with data available
Age	32.4 (11.4)	100%
Gender (Female)	35	100%
Minority	22	98%
Body mass index (n = 47)	25.9 (8.9)	77%
NTTI Skin spectrum (continuous)	36.6 (16.7)	100%
NTTI Skin type (categorical)		100%
Type I	*Excluded from PI-MED testing*	
Type II	17	
Type III	22	
Type IV	19	
Type V	3	
Type VI	0	
Composite erythema (non-transformed)		
Baseline composite erythema	2.13 (2.3)	100%
End-Point composite erythema (n = 43)	2.04 (2.0)	70%
Randomized to mindfulness intervention	35	100%
Baseline SHAPS anhedonia	2.11 (2.8)	100%
Baseline DARS overall anhedonia	48.1 (9.8)	100%
Baseline DARS SOCIAL ANHEDONIA	10.5 (3.2)	100%
Baseline PANAS positive affect	27.9 (7.6)	100%
Baseline PANAS negative affect	23.0 (7.2)	100%
Baseline perceived stress	22.1 (6.6)	100%
Baseline beck depression inventory II	15.7 (10.3)	100%

†Composite Erythema means and standard deviations were computed on untransformed variables.

### Ethical approval

This study was approved by the Virginia Tech IRB and all participants provided written informed consent in accordance with the Declaration of Helsinki.

## Measures

### PI-MED erythema

#### Precision implementation of minimal erythema dose (PI-MED)

Cutaneous erythema was induced and measured using PI-MED testing, a procedure recently adapted by Richey and colleagues^25^ to precisely measure individual differences in erythema response to ultraviolet, specifically UVB, light exposure. The PI-MED procedure is an adaptation of Minimal Erythema Dose (MED) testing^26^ intended to optimize the procedure for reproducibility and investigating individual differences in cutaneous inflammation. The PI-MED procedure should be considered relatively low-invasive as the erythema induced is similar to a mild/medium sunburn and is produced only within a few square inches of skin. MED, which is roughly equivalent to PI-MED in terms of participant UV-exposure, is a common procedure in dermatology.

#### Rationale for the PI-MED approach

MED testing is widely used in clinical contexts for determining UV-dosage for phototherapy. In typical MED procedures [see 26], the skin is exposed to different amounts of UVB (different time-based dosages), and erythema is determined by either a subjective judgement of whether there is “visible reddening” on the surface of the skin or is determined objectively using the a* scale from a spectrophotometer, which measures red hue. Using the objective spectrophotometer method, erythema or “burning” has been defined as an increase of at least 2.5 points on the post UV-exposure a* scale reading compared to the pre-exposure a* scale reading (i.e., Δa* >  = 2.5;^26^), and the ‘minimal erythema dose’ or MED, the output measure of typical MED testing, is defined as the *lowest exposure time* that produces ‘minimal erythema’ across several UV-exposure sites on the skin. While MED testing is widely used in clinical settings, a major limitation of typical MED testing for use in individual differences research is that the ‘minimal erythema dose’ is a discrete and rather coarse measure, necessarily limited in its variability by the timing intervals used across different UV-exposure sites. Further, traditional MED testing uses a binary cutoff for erythema. In contrast, PI-MED takes a different measurement approach, to provide a continuous and more direct measure of the full range of the erythema response. Where the MED is a measure of time, the outcome measure of PI-MED testing is an objective measure (via instrumentation) of the change in skin redness (Δa*) itself (instead of a binary erythema cutoff score), under a uniform UV intensity level and a UV dose intended to be standardized across skin pigmentation types. PI-MED’s advantage over traditional MED testing is that PI-MED captures continuous variation in the erythema response (from non-response through strong erythema), a measure that should be better suited for individual differences research.

#### Details of PI-MED standardization of UV-irradiation

In accordance with procedures described in detail by Richey and colleagues^25^, six small portions of skin on the non-dominant inner forearm were exposed, in sequence, to different temporal amounts of UV light, utilizing a novel dosage schedule^25^, calibrated to produce a reliable erythema response across different Fitzpatrick Skin Types^70^. The PI-MED dosage schedule is based on published median dosages^71^ required to produce a minimal erythema response for skin types II, III, and IV, with the dosage schedule for additional skin types extrapolated from these published medians [see 25 for further details]. The PI-MED procedure standardizes UV dosage with a constant UVB intensity of 270 μW/cm^2^, which is monitored via handheld radiometer. The novel dosage schedule, in combination with measurement of erythema via instrumentation (handheld spectrophotometer, described below) was designed to provide a closed loop measurement environment via temporal and energetic standardization of UV-irradiation across different skin types.

#### The PI-MED procedure

**Figure 1:**
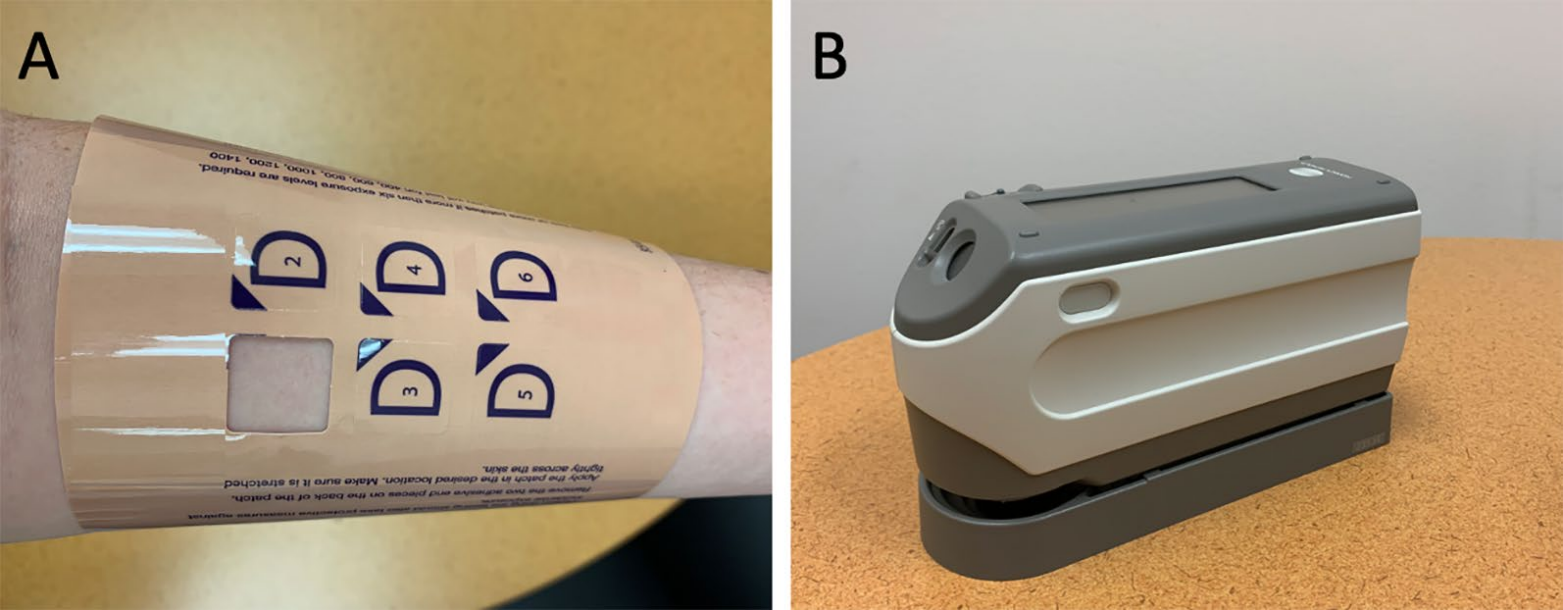
PI-MED Materials and Equipment. (**A**) six-aperture dose testing patch (purchased from The Daavlin Company) used to administer different UV dosages across six exposure sites on the skin. (**B**) handheld spectrophotometer (purchased from Konica Minolta) used to assess erythema by measuring skin redness.

Participants wore a six-aperture dose testing patch (purchased from^72^; see Fig. 1A) to allow different dosages of UV exposure across six sites on the skin. Aperture coverings were removed at timed intervals according to the published dosage schedule^25^. As UV-exposure is temporally reduced for each subsequently exposed site, PI-MED produces a gradated erythema response across the six exposure sites, such that the greatest erythema should occur in exposure site one and the least erythema response should occur in exposure site six. See Richey, and colleagues^25^ for additional details regarding materials and equipment.

#### Composite erythema measures

Erythema, or skin redness was measured via instrumentation both prior to UV-exposure and once again post-exposure at a follow-up session (conducted during a 24–48 h period when UVB-induced erythema is known to peak^73^). In accordance with prior work^26^, a handheld spectrophotometer (purchased from^74^; see Fig. 1B) was used to assess erythema by measuring skin redness. Specifically, the a* scale reading from each exposure site was used as an objective measure of both pre-exposure skin redness and post-exposure variation in skin redness induced by UV radiation. UV-induced erythema was computed by subtracting each exposure site’s pre-exposure a* scale reading from its post-exposure a* scale reading (Δa*). As detailed further in the section entitled ‘Assessment of PI-MED Reliability’, the Δa* measure was averaged across the six exposure sites to create a *Composite Erythema* measure, both for the study baseline time point (*Baseline Composite Erythema*) and for the study end-point (*End-Point Composite Erythema*). Primary analyses for the current study focus on the Baseline Composite Erythema measure. Internal consistency of both the Baseline Composite Erythema and the End-Point Composite Erythema measures were estimated across the respective six exposure sites’ Δa* measures. To examine changes in Composite Erythema across the whole study, *Studywise Composite Erythema* was computed by subtracting Baseline Composite Erythema from End-Point Composite Erythema.

#### Characterizing skin pigmentation

Degree of skin pigmentation is an important determinant of UV-induced erythema^38,75^. The Fitzpatrick Skin Type schema^70^ has been used extensively to classify human skin coloration on a scale of 0—40 points, which map onto six categories of skin pigmentation from lightest (I) to darkest (VI). In the context of MED testing, this schema has been used to determine the duration of UV exposure required to produce a minimal erythema response in an individual, based on their skin type [e.g., ^26^]. Broadly based on the Fitzpatrick schema, the skin-typing system developed by the National Tanning Training Institute^76^ classifies skin coloration on a continuous scale of 0—86 points (*NTTI Skin Spectrum)*, which map onto six primary skin type categories (*NTTI Skin Type:* I—VI). Previous research has validated the NTTI schema (Miller, et al., 2012), and compared to the Fitzpatrick system, the NTTI system offers a more fine-grained scale, assessing skin type based on: the color of untanned skin, hair and eye color, number of freckles, ethnic background, and sunburn and tan potential.

#### Calibration of PI-MED to skin type

The current study used the six primary NTTI Skin Type categories to determine UV dosage administration in accordance with the procedures and dosage schedule developed by Richey and colleagues^25^. Following recommendations of the NTTI instrument^76^ and protocols reported by Richey and colleagues^25^, PI-MED testing was not conducted on individuals with Type I skin, due to the potential for UV over-dosage. Calibrating the PI-MED dosage schedule to skin pigmentation based on categorical NTTI Skin Type (II-VI) is intended to attenuate differences in erythema response that would otherwise occur if different skin types were exposed to the same amount of UV.

#### Accounting for variation in PI-MED erythema not fully standardized by calibration to skin type

Although the PI-MED dosage schedule is calibrated to categorical NTTI Skin Type, there remains considerable between-individual variation in erythema response, possibly due to variation in the continuous NTTI Skin Spectrum score that is collapsed when using the categorical NTTI Skin Type. In other words, the continuous NTTI Skin Spectrum variable may more precisely approximate the skin’s intrinsic sensitivity to UV radiation beyond what is accounted for by the PI-MED dosage schedule. To assess the degree to which the continuous NTTI Skin Spectrum score affected PI-MED erythema response despite the calibration for categorical NTTI Skin Type (II-VI), the continuous 0—86 NTTI Skin Spectrum score was used as an additional covariate in analyses assessing associations between PI-MED erythema and other known inflammation covariates.

### Self-report measures of affect

Self-report measures of affect included: (A) two anhedonia measures: Snaith–Hamilton Pleasure Scale (SHAPS)^77^ and the Dimensional Anhedonia Rating Scale (DARS)^78^, which includes an overall anhedonia measure as well as a social anhedonia subscale; (B) measures of positive and negative affect from the Positive and Negative Affect Schedule (PANAS)^79^ (C) the Perceived Stress Scale (PSS)^80^ and (D) the Beck Depression Inventory (BDI-II)^81^. All measures and relevant subscales along with sample-specific internal consistency measures are detailed in the Supplemental Method.

### Non-affective covariates

Several non-affective measures collected at baseline previously associated with other measures of inflammation were also examined as covariates of PI-MED erythema including Age, Sex, Minority Status, and Body Mass Index [for review of inflammation covariates see 20]. It should be noted that previous research has not found age to be associated with MED and has inconsistently found MED to vary by sex^71,82^. Body mass index (BMI) was only collected for 77% of the sample (n = 47) at baseline, and analyses are presented both with and without this covariate to preserve power. As previously noted, we used the continuous NTTI Skin Spectrum score as a covariate to capture additional variance in erythema response beyond that induced by PI-MED dosage based on categorical Skin Type.

### Analytic approach

To examine preliminary validation of PI-MED testing for psychoneuroimmunology research, we examined (1) the standardization of PI-MED Composite Erythema across skin pigmentation types and the reliability of the PI-MED Composite Erythema measure and (2) associations between Composite Erythema and non-affective and affective covariates.

#### Assessment of PI-MED composite erythema standardization and reliability

As the specific dosage schedule implemented within PI-MED is novel to MED testing^25^ and was intended to standardize erythema response across different skin pigmentation types, we (1a) examined Composite Erythema across NTTI Skin Type using an ANOVA and follow-up t-tests, with the expectation that successful standardization of PI-MED erythema response would result in attenuated differences (ideally no differences) in Composite Erythema between the skin types. As there were only three subjects in the NTTI Skin Type IV group, this skin type was excluded from statistical analyses. Reliability of the PI-MED erythema measure was assessed by examining (1b) internal consistency across the six exposure sites (that were averaged to create both the Baseline Composite Erythema measure and the End-Point Composite Erythema measure). Specifically, Chronbach’s alpha was computed across the six Δa* values (spectrophotometer measures of pre-to-post UV exposure change in skin redness). PI-MED erythema reliability was also assessed in terms of (1c) the test–retest reliability of the Composite Erythema measures collected at baseline and at study end-point, approximately 8–10 weeks later. The test–retest reliability of Composite Erythema was examined in the whole sample, collapsing across the mindfulness and waitlist groups. Additionally, Composite Erythema test–retest reliability was also examined separately within the mindfulness and waitlist groups, as mindfulness interventions have previously been shown to lower inflammation^44,83,84^, suggesting that participation in the mindfulness intervention may alter test–retest associations.

#### Affective correlates of PI-MED composite erythema

As preliminary validation of PI-MED testing for psychoneuroimmunology research, we examined zero-order correlations between Composite Erythema and self-report measures of affect previously associated with peripheral inflammation markers, including measures of anhedonia (detailed below), PANAS Positive and Negative Affect, the Perceived Stress Scale, and the Beck Depression Inventory. Given research suggesting that inflammation may be particularly associated with symptoms of anhedonia, we hypothesized that baseline anhedonia measures, specifically, SHAPS Anhedonia, DARS Overall Anhedonia, and DARS Social Anhedonia, would show associations with Composite Erythema, beyond the effects of non-affective covariates. To test this, we estimated hierarchical regressions in which different anhedonia measures were (each separately) added to base prediction models with non-affective covariates including NTTI Skin Spectrum, Sex, Age, Minority Status, and Body Mass Index.

**Figure 2:**
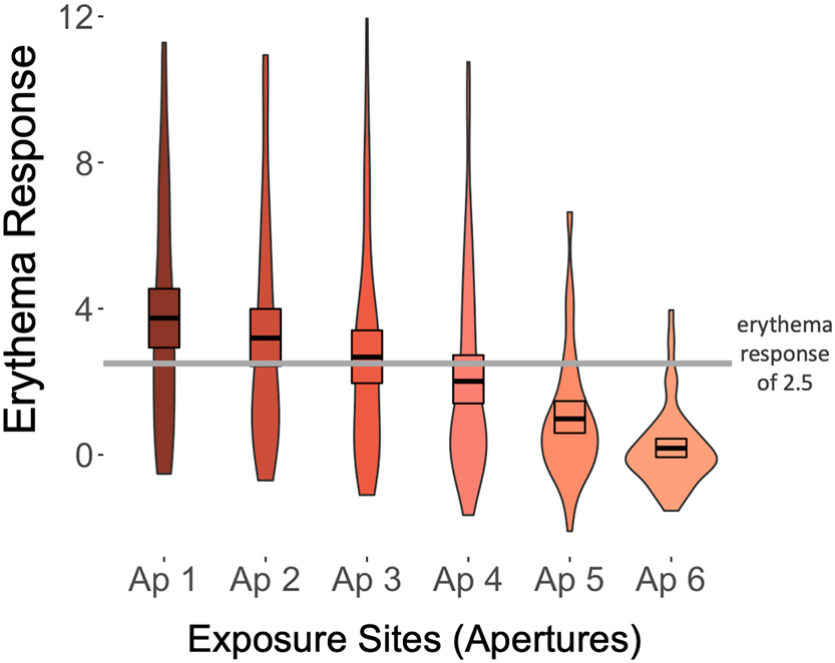
Violin plots summarizing means, 95% CIs, and distributions for the (raw, non-transformed) erythema responses within each exposure site. Raw erythema showed a linear response across the six exposure sites, collapsing across all skin types. The six exposure sites represent the given UV dosage for the given skin type, according to the PI-MED dosage schedule. The grey horizontal line marks the threshold for erythema as defined by Heckman and colleagues^26^.

In the Supplement we also present multi-level models that estimated erythema response across the six aperture measures (dosage), nested within person, along with affective and non-affective covariates. However, we chose to focus the main manuscript on models estimating Composite Erythema (rather than individual aperture measures), in order to collapse across any potential systematic measurement error between the odd and even numbered apertures/exposure sites (see Fig. 2 from^25^). While the hierarchical regressions in the main manuscript were estimated using the log-transformed Composite Erythema scores as the outcome measure, in the Supplement we present a duplicate series of multi-level models that were estimated using (a) the raw erythema scores across the six apertures and (b) the log-transformed erythema scores across the six apertures. Additionally, prospective associations between baseline anhedonia measures and End-Point Composite Erythema are presented in the Supplement.

## Results

### Participant characteristics

The baseline sample was comprised of 61 participants (35 female; 22 ethnic minority: 11 Asian, 3 Black/African American, 1 Hispanic, 4 Middle Eastern, 3 other), ranging in age from 18—57 (M = 32.4, SD = 11.4). Following baseline data collection, approximately half of the participants (n = 35) were randomized to the mindfulness intervention while the remaining participants were placed on the waitlist. The average BMI for the sample was 25.9 (SD = 8.9). The sample included NTTI Skin Types II—V, and consistent with self-reported demographics, 64% of participants fell within the lighter skin tone categories of Type II and III. Baseline self-reported stress levels on the Perceived Stress Scale (M = 22.1, SD = 6.6) were substantially higher than previously reported population norms^80^, confirming that our sample was, in line with the clinical trial recruitment strategy, indeed comprised of highly stressed individuals. See Table 1 for sample demographics and other variables of interest.

### Manipulation check: sensitivity of erythema response to PI-MED dosages

As expected, across the six exposure sites, PI-MED-induced erythema showed a linear response to the novel dosage schedule (see Fig. 2 and Supplemental Results). Using the definition of erythema (Δa* >  = 2.5) detailed by Heckman and colleagues^26^, 24 of our 61 (39%) participants did not show erythema in any exposure site and 37 of 61 (61%) showed erythema in at least one expoosure site. However, it should be noted again that PI-MED’s continuous measure of erythema response was the variable of interest rather than the traditional MED binary cutoff score. There was not a significant difference in the NTTI Skin Spectrum scores between those who did (M = 34.1; SD = 16.0) and did not (M = 40.4; SD = 17.2) show an erythema response (*t*(47) = 1.4, p = 0.157).

### Standardization of PI-MED erythema

**Figure 3:**
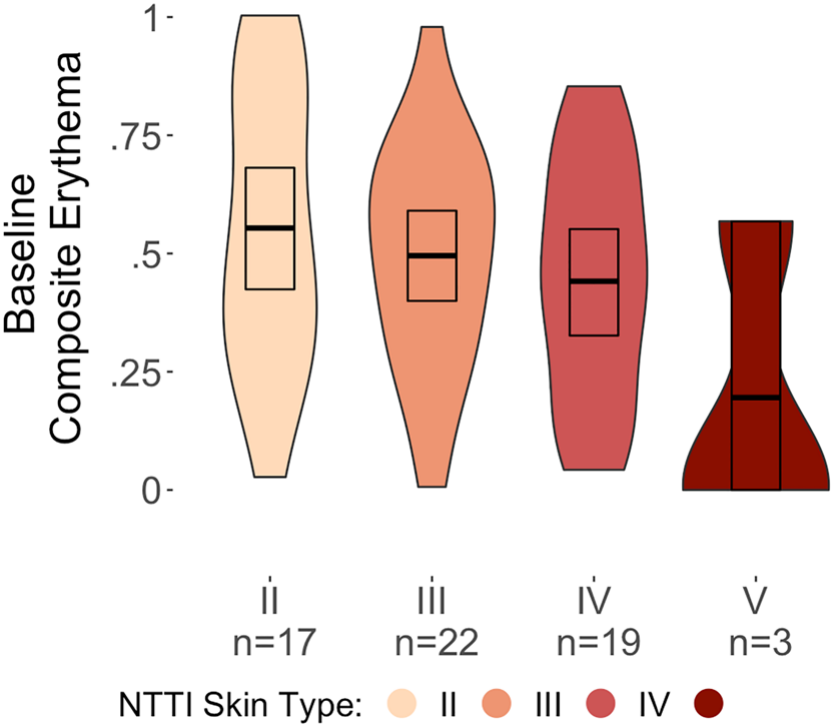
Violin plots summarizing means, 95% CIs, and distributions for Baseline Composite Erythema across NTTI Skin Types. In line with PI-MED’s intended standardization of UV dosage across skin types, differences in Composite Erythema between NTTI Skin Types II, III, and IV appeared to be relatively minimal and were non-significant. However, average Composite Erythema for NTTI Skin Type V was substantially lower than that of the other skin types.

The means and distributions for Baseline Composite Erythema across NTTI Skin Types are depicted in Fig. 3. The effect of NTTI Skin Type (excluding Type V) on Composite Erythema was not significant [F(2,55) = 0.83, *p* = 0.44]. Follow-up t-tests, conducted for robustness, did not reveal any significant differences in Composite Erythema between either NTTI Skin Type II (M = 0.55, SD = 0.30) and Type III (M = 0.50, SD = 0.24; *t*(37) = 0.68, *p* = 0.50), or between NTTI Skin Type II and Type IV (M = 0.44, SD = 0.25; *t*(34) = 1.23, *p* = 0.23), or between NTTI Skin Type III and Type IV (*t*(39) = 0.71, *p* = 0.48). A lack of significant differences here is consistent with the PI-MED dosage schedule standardizing the erythema response across Skin Types II–IV. While formal statistical analysis could not include NTTI Skin Type V due to the small number of individuals in the group, the mean Composite Erythema for this group was substantially lower (M = 0.20, SD = 0.32) than that of the other skin types.

To assess the degree to which the continuous NTTI Skin Spectrum score affected PI-MED erythema response despite the PI-MED dosage calibration to categorical NTTI Skin Type, associations between NTTI Skin Spectrum and Composite Erythema were estimated (zero-order correlations) across the whole sample and were found to be moderate at both baseline (*r* = − 0.26, 95% CI = [− 0.48, − 0.01]) and at end-point (*r* = − 0.35, 95% CI = [− 0.59, − 0.05]; see Table 2).

**Table 2:** Relationships between Composite Erythema and covariates.

	Baseline composite erythema				
	*r*	95% CI	df		
**Correlations between baseline erythema and known inflammation covariates**					
**NTTI skin spectrum**	**− .26**	**[− .48, − .01]**	59		
Age	− .16	[− .39, .10]	59		
Body mass index	− .09	[− .37, .20]	45		
**Baseline SHAPS anhedonia**	**.27**	**[.02, .49]**	59		
Baseline DARS overall anhedonia	− .23	[− .46, .02]	59		
**Baseline DARS social anhedonia**	**− .29**	**[− .50, − .04]**	59		
Baseline PANAS positive affect	− .14	[− .38, .11]	59		
Baseline PANAS Negative affect	− .05	[− .30, .20]	59		
Baseline perceived stress	.15	[− .11, .39]	59		
Baseline beck depression inventory II	.13	[− .13, .37]	59		

	Baseline composite erythema				
	mean diff	*t*	95% CI	Cohen's d	df
**Between- group differences in baseline composite erythema**					
Female vs. male	− .03	− .48	[− .17, .11]	− .13	59
Minority vs. white	− .14	− 1.90	[− .28, .00]	− .52	58
Mindfulness intervention vs. waitlist	.02	.35	[− .12, .16]	.09	59

	End-Point composite erythema—baseline composite erythema				
	mean diff	*t*	95% CI	Cohen's d	df
**Between-group differences in studywise change in erythema response**					
Female vs. male	.04	.66	[− .07, .15]	.20	41
Minority vs. white	− .02	− .41	[− .15, .10]	− .14	40
Mindfulness intervention vs waitlist	.05	.90	[− .06, .16]	.27	41

Significant values are in bold.

## Reliability of PI-MED erythema

### Internal consistency of composite erythema

All six Δa* values were available for each subject who completed the PI-MED procedure at each time point (i.e., within the PI-MED sessions, there was no missing data across the six exposure sites). The Composite Erythema measure demonstrated excellent internal consistency across the six exposure sites for both the Baseline Composite Erythema (α = 0.94, n = 61) and for the End-Point Composite Erythema (α = 0.94, n = 43) measures.

### Test–retest reliability of composite erythema

**Figure 4:**
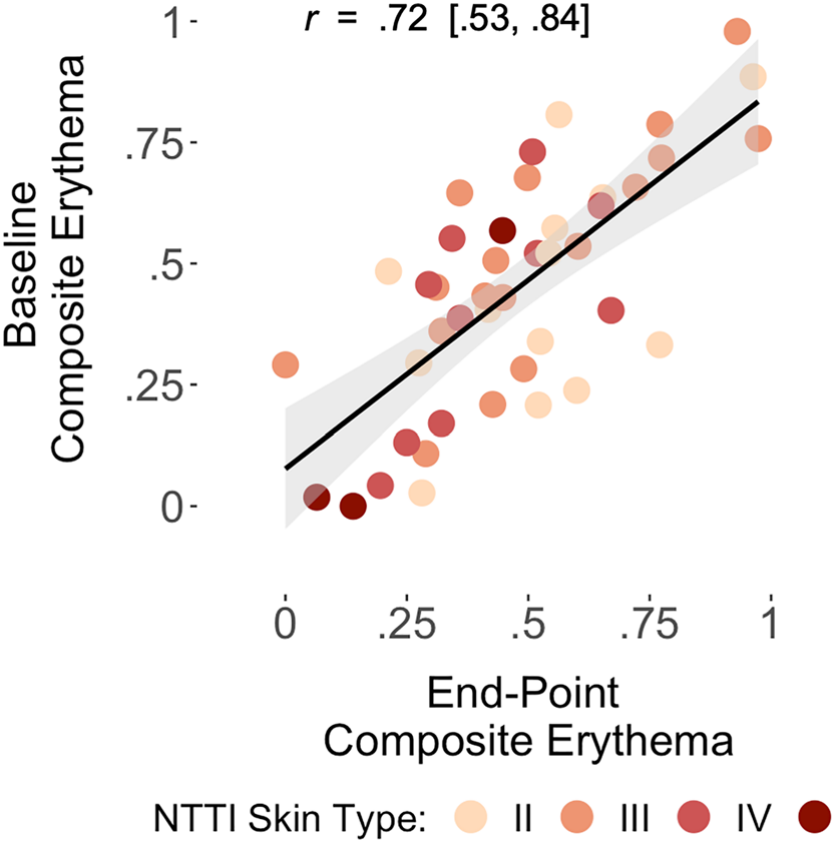
Scatterplot depicting the re-test reliability for Composite Erythema. PI-MED-induced Composite Erythema showed good test–retest reliability between the baseline and end-point measurements (*r* = .72, 95% CI = [.53, .84]). The four skin types included in the sample appeared well-distributed, suggesting the PI-MED procedure reliably induced erythema across different skin pigmentation types.

The Composite Erythema measures were highly skewed both at baseline (skew = 1.1) and at end-point (skew = 1.55), and thus both variables were log-transformed for subsequent analyses. The Composite Erythema measure showed good test–retest reliability between the baseline and end-point assessments (n = 43; *r* = 0.72, 95% CI = [0.53, 0.84]; see Fig. 4). Further, the four skin types represented in the current sample appeared to be well distributed within the association between the baseline and end-point measures of Composite Erythema (see Fig. 4). In the mindfulness group, Composite Erythema test–retest was excellent (n = 23; *r* = 0.79, 95% CI = [0.56, 0.91]), while in the waitlist group, Composite Erythema test–retest was relatively attenuated (n = 20; *r* = 0.58, 95% CI = [0.19, 0.82]).

## PI-MED erythema covariates

Zero-order correlations between baseline covariates and Baseline Composite Erythema and between-group differences in both Baseline Composite Erythema and Studywise Composite Erythema are presented in Table 2. As hypothesized, anhedonia measures showed significant moderate associations with Baseline Composite Erythema, with Baseline SHAPS Anhedonia showing a positive association (*r* = 0.27, 95% CI = [0.02, 0.49]) and Baseline DARS Social Anhedonia showing a negative association (*r* = −0.29, 95% CI = [ −0.50,  −0.04]). While the DARS Overall Anhedonia measure showed an association in a consistent direction, the association was not significant and none of the other affective covariates showed significant associations with Baseline Composite Erythema. As the SHAPS and DARS measures were coded in opposite directions (higher values representing greater anhedonia and lesser anhedonia, respectively), there was a consistent pattern of increased anhedonia associated with a greater erythema response. NTTI Skin Spectrum showed a significant negative association with Baseline Composite Erythema such that greater skin pigmentation was associated with less erythema. None of the other affective or non-affective covariates showed significant associations with Baseline Composite Erythema, and there were no significant between-group differences in either the Baseline Composite Erythema nor the Studywise Composite Erythema measure for sex, minority status, or randomization to the mindfulness intervention.

## Concurrent associations between anhedonia and PI-MED erythema

Models examining concurrent associations between Baseline Composite Erythema and baseline predictors including demographic variables and NTTI Skin Spectrum are presented in Table 3. A base model including all covariates accounted for 9% of the variance and was not significant, possibly due to the smaller sample (n = 46) produced from including the BMI variable (which was available for only 77% of the sample). A base model excluding BMI (n = 60) was nearly significant (*p* = 0.06), accounted for 15% of the variance, and was determined to be the Final Base Model. In this model, only age was a significant (negative) predictor of Baseline Composite Erythema (β = − 0.29, *p* = 0.03).

**Table 3:** Concurrent associations between anhedonia measures and PI− MED erythema.

	β Coef	B Coef	SE	t	*p*	95% CI
**Base model with all covariates (n = 46)**	**F(5,40) = .80, *p* = .56, R2 = .09, Adj- R2 = − .02, SE of Estimate = .27**					
**Constant		.85	.22	3.86	< .001	[.41, 1.30]
NTTI skin spectrum	− .19	.00	.00	− .67	.51	[− .01, .01]
Sex	− .11	− .06	.08	− .71	.48	[− .23, .11]
Age	− .20	− .01	.00	− 1.16	.25	[− .01, .00]
Minority	− .10	− .05	.16	− .32	.75	[− .38, .27]
Body mass index	− .05	.00	.00	− .31	.76	[− .01, .01]
~ *Final base model (n = 60)	**F(4,55) = 2.40, *p* = .06, R2 = .15, Adj-R2 = .09, SE of Estimate = .26**					
**Constant		.89	.16	5.47	< .001	[.56, 1.21]
NTTI skin spectrum	− .23	.00	.00	− 1.02	.31	[− .01, .00]
Sex	− .07	− .04	.07	− .52	.61	[− .17, .10]
*Age	− .29	− .01	.00	− 2.16	.03	[− .01, − .00]
Minority	− .16	− .09	.12	− .72	.47	[− .34, .16]
*Hierarchical model: final Base + SHAPS (n = 60)	**﻿ΔF(1,54) = 4.78, *p* = .03, R2 = .22, Adj-R2 = .15, ΔR2 = .07, SE of Estimate = .25**					
**Constant		.84	.16	5.30	< .001	[.52, 1.16]
NTTI skin spectrum	− .36	− .01	.00	− 1.61	.11	[− .01, .00]
Sex	− .05	− .03	.07	− .40	.69	[− .16, .11]
~ *Age	− .24	− .01	.00	− 1.87	.07	[− .01, .00]
Minority	− .01	.00	.13	− .03	.97	[− .26, .25]
*Baseline SHAPS anhedonia	.28	.03	.01	2.19	.03	[.00, .05]
~ *Hierarchical model: final base + DARS overall (n = 60)	**﻿ΔF(1,54) = 3.84, *p* = .06, R2 = .21, Adj-R2 = .13, ΔR2 = .06, SE of Estimate = .25**					
**Constant		1.34	.28	4.79	< .001	[.78, 1.91]
NTTI Skin Spectrum	− .25	.00	.00	− 1.17	.25	[− .01, .00]
Sex	− .03	− .02	.07	− .24	.81	[− .15, .12]
*Age	− .29	− .01	.00	− 2.23	.03	[− .01, − .00]
Minority	− .13	− .07	.12	− .58	.57	[− .32, .17]
~ *Baseline DARS overall anhedonia	− .24	− .01	.00	− 1.96	.06	[− .01, .00]
*Hierarchical model: final base + DARS social (n = 60)	**﻿ΔF(1,54) = 6.75, *p* = .01, R2 = .24, Adj-R2 = .17, ΔR2 = .09, SE of Estimate = .25**					
**Constant		1.21	.20	6.11	< .001	[.81, 1.61]
NTTI Skin Spectrum	− .33	− .01	.00	− 1.55	.13	[− .01, .00]
Sex	− .04	− .02	.07	− .30	.76	[− .15, .11]
*Age	− .29	− .01	.00	− 2.27	.03	[− .01, − .00]
Minority	− .05	− .03	.12	− .23	.82	[− .27, .22]
*Baseline DARS Social Anhedonia	− .32	− .03	.01	− 2.60	.01	[− .05, − .01]
**p < .01						
*p < .05						
~ *p ≤ .07						

**Figure 5:**
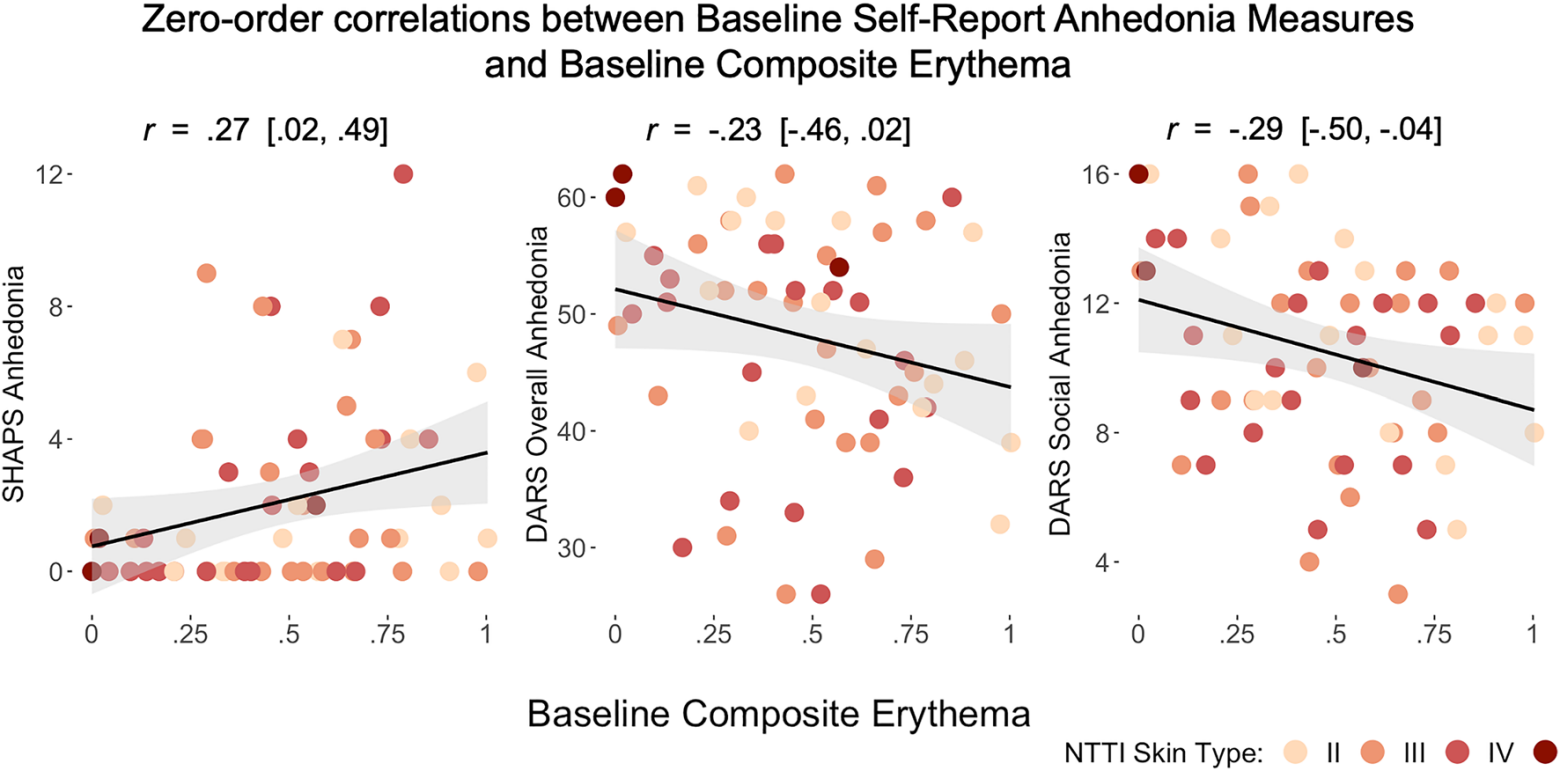
Scatterplots depicting the zero-order associations between Baseline Composite Erythema and each of the baseline anhedonia measures.

To examine concurrent associations between baseline anhedonia measures and Baseline Composite Erythema above and beyond non-affective covariates, a hierarchical model was estimated for each of the three anhedonia measures, with each added separately to the Final Base Model. The model that added Baseline SHAPS Anhedonia significantly improved upon the Final Base Model (*p* = 0.03) and accounted for an additional 7% of the variance (R2 = 0.22), with Baseline SHAPS Anhedonia as a significant positive predictor of Baseline Composite Erythema (β = 0.28, *p* = 0.03). The model that added Baseline DARS Overall Anhedonia was a near-significant improvement upon the Final Base Model (*p* = 0.06), accounting for an additional 6% of the variance (R2 = 0.21) compared to the Final Base Model. The model that added Baseline DARS Social Anhedonia significantly improved upon the Final Base Model (*p* = 0.01) and accounted for an additional 9% of the variance (R2 = 0.24), with Baseline DARS Social Anhedonia as a significant negative predictor of Baseline Composite Erythema (β = − 0.32, *p* = 0.01). For illustrative purposes, the zero-order correlations between Baseline Composite Erythema and each of the baseline anhedonia measures is shown in Fig. 5.

In the series of multi-level models estimating the separate effects of different affective covariates (in addition to non-affective covariates) on the log-transformed erythema response across each of the six PI-MED exposure sites (see Supplemental Results and Supplemental Tables 1, 2, 3, 4, 5, 6, 7), the only affective covariates to show significant effects on erythema were Baseline SHAPS Anhedonia (*p* = 0.038) and Baseline DARS Social Anhedonia (*p* = 0.013), while Baseline DARS Overall Anhedonia (*p* = 0.055) and Baseline PANAS Positive Affect (*p* = 0.092) had “trend-level” effects. No other affective predictors showed significant or near-significant associations (*p*’s > 0.10), suggesting the relationship between erythema and affect may be specific to the domain of positive emotion. When each of the three baseline anhedonia measures was tested separately in multi-level models that included all other affect predictors (see Supplemental Tables 8, 9, 10), only Baseline DARS Social Anhedonia had a “trend-level” effect on the transformed erythema scores (*p* = 0.055), while no other affect predictors in the model had significant effects.

In the prospective models (see Supplemental Results), only SHAPS Anhedonia was a significant improvement upon a base model in prospective prediction of PI-MED erythema; however, the direction of the association was reversed with greater anhedonia predicting lower erythema at study endpoint. These results likely reflect regression to the mean and are discussed further in the Supplement.

## Discussion

Psychoneurocutaneous research is a burgeoning area^1,2^, but understanding relationships between affect and skin inflammation is limited by the lack of an established protocol for measuring cutaneous inflammation that is suitable for individual differences research. To address this gap, we present a preliminary validation of Precision Implementation of Minimal Erythema Dose, or PI-MED testing^25^ as a method for measuring cutaneous inflammation and further demonstrate its relevance for psychoneuroimmunology research. Specifically, we demonstrate that PI-MED’s novel UV dosage schedule produces a reasonably standardized erythema response across NTTI Skin Types II–IV and that PI-MED erythema shows strong internal consistency across UV-exposure sites and good test–retest reliability across 8–10 weeks. Together, this evidence supports the use of PI-MED as a protocol for inducing and measuring individual differences in cutaneous inflammation. Additionally, we examined associations between PI-MED erythema and known covariates of other measures of peripheral inflammation and, unexpectedly, found that most non-affective and affective covariates showed only weak and/or non-significant associations. However, as predicted, cutaneous erythema response demonstrated a specific relationship with anhedonia, beyond associations with non-affective covariates.

The novel contribution of PI-MED is the focus on reproducibility, achieved through the application of a uniform level of UV intensity (270 μW/cm^2^), temporal standardization of UV dosage across different skin types, and objective measurement of the full range of erythema response via spectrophotometer. The lack of a significant effect of NTTI Skin Type (for Types II–IV) on PI-MED erythema in models including other covariates can be considered preliminary evidence of PI-MED having a standardizing effect on cutaneous erythema response across different skin pigmentation types. However, as PI-MED is a recently adapted procedure that yielded a novel measure of inflammation, we did not conduct a power analysis, and thus, null findings can only provide limited evidence for erythema standardization. Further, evidence for erythema standardization is limited to Skin Types II–IV (discussed further below). Despite evidence of standardization across skin types, NTTI Skin Spectrum scores showed a moderate, significant zero-order correlation with PI-MED erythema, likely due to additional variation not accounted for by calibrating PI-MED dosage to skin type categories. The composite PI-MED erythema measure showed good internal consistency within person across six exposure sites at both baseline and at study end-point. Further, the composite PI-MED erythema measure showed good test–retest reliability across an 8–10-week interval. Importantly, the four different skin types included in the current study appeared to be well distributed within the association between the baseline and end-point composite erythema measures, suggesting erythema was reliably induced across skin types within the sample.

Across three anhedonia measures, higher baseline anhedonia levels showed consistent concurrent associations with increased baseline erythema, controlling for non-affective predictors. Interestingly, concurrent associations between anhedonia and PI-MED erythema were of a similar magnitude to associations between erythema and NTTI Skin Spectrum score, suggesting that the relationship between anhedonia and cutaneous erythema is not trivial, in line with other research suggesting a particularly strong relationship between inflammation and anhedonia^17,45–50^. Further, in the full multi-level models, only social anhedonia showed a near-significant association with erythema beyond the effects of the other affective predictors. Together with the relatively weak and non-significant associations between erythema and perceived stress, depression, and negative affect, it appears that UV-induced erythema may show a discriminate association with anhedonia and with social anhedonia.

While evidence supports a distinct relationship between inflammation and symptoms of anhedonia, other evidence calls into question the specificity and scope of this association. For example, considerable evidence suggests inflammation may map onto the atypical subtype of depression—characterized by interpersonal rejection sensitivity, mood reactivity, greater prevalence of childhood maltreatment, hypersomnia, and increased appetite—more closely than it maps onto the melancholic subtype—characterized by anhedonia, diminished mood reactivity, reduced sleep, and reduced appetite^85^, which suggests that the association between anhedonia and inflammation might not be particularly strong relative to other symptom profiles. On the other hand, symptom profiles from data-driven subtypes of depression do not fully align with the clinically characterized subtypes and inflammation (specifically TNFα levels) was similarly predictive of both atypical and non-atypical subtypes^86^. Further, in a large nationally representative US sample, when atypical depression was defined only by the presence of a major depressive episode plus hypersomnia and hyperphagia, a higher percentage of those with atypical than non-atypical depression endorsed symptoms of anhedonia^87^. Further, childhood maltreatment, which is associated more commonly with the atypical subtype, is itself linked to reduced reward responsivity^88^, lower positive affect^89^, greater anhedonia^90^ and increased inflammation ^91,92^, see^93^ for review]. Together, these findings point to the need for improved clinical phenotyping and for understanding specific inflammatory processes in relation to specific transdiagnostic symptoms.

Another interesting challenge to the specificity of the association between inflammation and anhedonia is experimental work in humans showing that induced inflammation *increases* reward-related neural activity in response to viewing close others^94^ and in response to positive social feedback^95^. These findings must be interpreted in the broader context of induced inflammation also increasing neural sensitivity to negative social feedback^95^ and increasing feelings of social disconnection^96^. Together, these results can be explained in terms of inflammation producing “sickness behavior” in which inflamed individuals are in a vulnerable state and show a corresponding increase in sensitivity to social stimuli, including greater threat-related processing, and enhanced approach motivation towards close and friendly others who may provide support^67^. It is possible that high levels of social sensitivity may produce anhedonia-related sequelae^97^, and future work should investigate this possibility. Indeed, understanding the dimensionality of anhedonia and its association to reward responses to different classes of reward is open area of inquiry [e.g.,^98^], and future work should aim to map specific inflammatory processes to dimensions of anhedonia and reward responsivity.

## Limitations

The current study has some important limitations that constrain generalization of findings and should guide future work. First, associations between PI-MED erythema and most potential covariates were not fully consistent with previous findings. For example, while previous research has not found an association between age and MED, a distinct but related measure to PI-MED erythema^71,82^, the current study found a significant negative association between age and PI-MED erythema. Future work should seek to firmly establish how age may be related to erythema response. Whether age is indeed negatively associated or not associated with erythema—either finding would constitute a potentially advantageous feature of the PI-MED procedure compared to existing measures of inflammation. Given that age shows robust positive associations with a number of pro-inflammatory markers measured via blood^20^, PI-MED erythema’s divergent age-related profile would make this novel method an excellent complementary assay that could, in conjunction with existing assays, potentially shed light on how different inflammatory processes may be associated with developmental processes. Relationships between erythema and other potential covariates were relatively weak and non-significant, raising questions about how PI-MED erythema relates to peripheral inflammation markers. Future work should examine PI-MED erythema in relation to other markers of systemic inflammation (e.g., plasma IL-6), bearing in mind that previous work has found weak and inconsistent associations between inflammatory markers across different measurement methods^19,22^. When used in conjunction with existing assays of inflammation, PI-MED may elucidate whether cutaneous inflammatory processes may diverge from inflammatory processes in the periphery more generally.

Another limitation is that evidence of PI-MED’s standardization of the erythema response is limited to Skin Types II–IV, due to excluding Skin Type I from the procedure, and under-sampling or no sampling, respectively, of Types V and VI. Further, as Skin Type V showed a much lower erythema response compared to other skin types, it suggests the current PI-MED dosage schedule may be inappropriately calibrated for the darkest skin types. This is consistent with previous research using the MED measure that found large differences in erythema sensitivity between Skin Types V and VI and lighter skin types^38,99^. Thus, future work should continue to refine the PI-MED dosage schedule to produce a similar erythema response across different skin types, with particular attention to calibrating the dosage for the darkest (Types V and VI) and lightest (Type 1) skin types. Together with research suggesting that assessment of skin type via self-report is biased by Eurocentric White norms^100^, future work should particularly focus on refining both skin type assessment and UV dosage schedules to ensure procedures are appropriate for dark-skinned people. Further, the PI-MED dosage schedule was based on extrapolating previously published median MED results for lighter skin types; however, future work might consider altering the dosage schedule such that other functional forms are assumed to underlie the association between skin type and dosage time, with relatively lower dosages for Type I and relatively higher dosages for Types V and VI.

Finally, the current study has only examined a subset of important parameters related to PI-MED erythema. While we did demonstrate the sensitivity of PI-MED erythema to the novel dosage schedule, its test–retest reliability, and its association with the theoretically-relevant construct of anhedonia, the current study was likely underpowered to detect potential between-group differences (e.g., between the mindfulness intervention and wait-listed groups). Future efforts to demonstrate construct validity could assess whether the PI-MED measure shows between-group differences in inflammatory response or sensitivity to other experimentally induced changes, as has been previously demonstrated with MED, the traditional, but less fine-grained, measure of erythema [e.g.,^35^]. Furthermore, the current study was not designed to assess causal directionality of associations between anhedonia and erythema. Future work might address questions related to how affective processes and erythema may bi-directionally influence one another using ecological momentary assessment and potentially more substantial erythema challenges (beyond PI-MED’s induction of erythema on only a few square inches of skin).

## Advantages of PI-MED for assessing inflammatory tone

The PI-MED procedure offers several advantages for measuring inflammatory tone beyond existing blood-based and salivary protocols. First, there is potential for reduced cost of assessment. Investigations of inflammatory markers drawn from peripheral fluid may cost ~ $10 in ELISA kits and ~ 6 h in technician time, per protein, per person as well as ~ 0.5 h in phlebotomist time, per person^101^, with no particular savings across multiple measurement sessions. The cost for a study examining five inflammatory proteins with 100 subjects at two time points would be ~ $10,000 in ELISA kits alone. In comparison, the primary expenses involved in PI-MED are the up-front costs of the spectrophotometer (~ $8,300), UVB lamp (~ $300), and handheld radiometer (~ $200), with the costs for multiple assessments amounting to relatively negligible costs for dose testing patches and replacing UV bulbs over time. Further, procedural training and staff time required to administer PI-MED are relatively low; research assistants can be trained to administer PI-MED within several hours, and the PI-MED procedure can be completed in less than an hour plus a 15 min follow-up appointment, per person. Thus, as participant numbers and number of repeated assessments increases, PI-MED can offer considerable savings over blood-based and salivary inflammatory assays.

Another advantage of PI-MED is that it offers a biologically-valid measure of inflammatory tone as well as inflammatory response in the skin. While existing blood-based and salivary protocols aim to measure inflammation that is clinically meaningful, costs often limit these studies such that only a few specific inflammatory proteins are examined, leaving unanswered questions about how such individual inflammatory markers act in coordination, in vivo. An alternative approach employed for measuring inflammation from peripheral fluids is to create a composite measure by averaging values from several proteins; however, such composite measures suffer from relatively poor physiometrics and issues with the biological plausibility of composites as a meaningful measure of inflammatory tone^102^. In contrast, PI-MED-induced erythema is a biologically-valid measure that results from a multi-component coordinated response to UVB inflammatory challenge. PI-MED offers real-world validity as the UVB inflammatory challenge is analogous to exposure from the sun, a common environmental stressor. In addition to offering a specific measure of inflammation in the skin, evidence suggests the cutaneous response is influenced by systemic factors [e.g.,^32^, ^34–37^], thus suggesting PI-MED is also an indirect measure of general inflammatory tone.

## Conclusions

While further refinement for broader skin types is needed, PI-MED offers a promising method for inducing and measuring individual differences in cutaneous inflammation that can serve as a complementary or alternative measure to blood-based and salivary protocols. Further, PI-MED offers opportunity to study relationships between cutaneous inflammation and inflammation markers sampled from fluids and between cutaneous inflammation and affect, particularly anhedonia-related symptoms. Overall, PI-MED has potential to expand psychoneuroimmunology research for better understanding immunological and affective processes in relation to the skin.

## Supplementary Information

Supplementary Information.

## Data Availability

The datasets analysed during the current study are available in the Open Science Framework (OSF) repository, https://osf.io/yh458/wiki/Erythema%20Data/.
