# Supplementary material for: Validation of a novel method of ultraviolet-induced cutaneous inflammation and its associations with anhedonia
Source: Sci Rep. 2022 Nov 24;12:20237. doi: 10.1038/s41598-022-24598-4 (PMC9691739; doi:10.1038/s41598-022-24598-4)
Supplement: Supplementary file 1 — Supplementary Information. [file 41598_2022_24598_MOESM1_ESM.docx]

**Supplemental Method**

***Measuring*** ***Prospective Change in PI-MED Erythema.*** We also examined prospective prediction of increases in Composite Erythema response across the study period, beyond the effects of non-affective covariates. For these longitudinal analyses using erythema measures from both baseline and the study end-point, randomization to the mindfulness intervention was also included as a binary predictor (mindfulness vs. waitlist), as mindfulness interventions have previously been found to lower peripheral inflammation (Rosenkranz, et al., 2013; Sanada, et al., 2020; Villalba, et al., 2019). Further, as previously reported in the current sample (Carlton, et al., 2021), those that participated in the mindfulness intervention showed reduced social anhedonia, a core construct of interest in the current study.

***Self-Report Measures of Affect***

**Snaith–Hamilton Pleasure Scale (SHAPS).** The 14-item SHAPS (Snaith, et al., 1995) was used to measure self-reported anhedonia over the past several days. In line with typical procedures, scoring for each item was binarized: collapsing both “strongly agree” and “agree” to a score of 0 and collapsing both “strongly disagree” and “disagree” to a score of 1. All items were summed to create the total *SHAPS Anhedonia* variable (score range 0—14), with higher scores representing greater anhedonia. In the current sample, *SHAPS Anhedonia* showed good internal consistency (α = .83).

﻿**Dimensional Anhedonia Rating Scale (DARS).** The 17-item DARS (Rizvi et al., 2015) assesses self-reported anhedonia across 4 domains: hobbies/past-times, food/drinks, social activities, and sensory experiences. Within each domain, participants provide at least two examples of things they find rewarding/pleasurable. Then, referencing their provided examples in each domain, participants made ratings on 5-point Likert-type scale ranging from 0 (“not at all”) to 4 (“very much”), indicating their desire and motivation for, effort to obtain, and pleasure associated with their given domain examples. Higher scores indicate increased desire and motivation for, effort to obtain, and pleasure (i.e., less anhedonia). For the current study, we were interested in the 4-item *DARS Social Anhedonia* subscale and the *DARS Overall Anhedonia* scale (summing across 4 domains). In the current sample, *DARS Overall Anhedonia* showed good internal consistency (α = .83) and *DARS Social Anhedonia* showed acceptable internal consistency (α = .76).

**Positive and Negative Affect Schedule (PANAS).** General valanced affect was measured using the 10-item PANAS Positive Affect subscale and the 10-item PANAS Negative Affect subscale (Watson & Clark, 1999). Participants indicated how often they “generally” experienced different affect states during the past few weeks on a 5-point Likert-type scale. ﻿Response items ranged from 1 (“very slightly or not at all”) to 5 (“extremely”), with higher scores representing more intense levels of emotion. In the current sample, *PANAS Positive Affect* showed good-to-excellent internal consistency (α = .90) and *PANAS Negative Affect* showed good internal consistency (α = .86).

﻿**Perceived Stress Scale (PSS).** The 10-item PSS (Cohen, 1988) measured perceived stress over the past month with participants rating how often they felt a certain way (e.g., “In the last month, how often have you felt confident about your ability to handle your personal problems”) on a 5-point Likert-type scale ranging from 0 (“never”) to 4 (“very often”), with higher scores representing greater perceived stress. In the current sample, *Perceived Stress* showed good internal consistency (α = .88).

﻿ ﻿**Beck Depression Inventory (BDI-II).** The 21-item BDI-II (Beck, et al., 1996) assessed the severity of depressive symptoms over the past 2 weeks, with participants endorsing different depression symptoms on a 4-point Likert-type scale ranging from 0 to 3, with higher scores indicating greater severity of depressive symptoms. In the current sample, *Beck Depression Inventory* *II* showed excellent internal consistency (α = .91).

**Supplemental Results**

***Multi-Level Models.*** All multi-level models estimated erythema response across the six exposure site measures (representing dosages), nested within person, with person modeled as a random effect. The base model also included NTTI Skin Spectrum, Age, Sex, and Minority Status as fixed between-person predictors. All models were estimated for both (1) the raw erythema response and (2) the log-transformed erythema response (see Supplemental Tables 1-10). Models that added random slopes (for exposure site) did not converge. In all models, there was a significant fixed effect of exposure site (all *p* < .001), demonstrating that the group-level erythema response was sensitive to the PI-MED dosage manipulation and the raw erythema data showed a linear response across the six dosages (see Main Manuscript Figure 1). Further, in all models there was a significant negative effect of age, such that the erythema response decreased with age. Separate models (see Supplemental Tables 1-7) added each of the affect predictors individually to the base model as fixed effects.

In this series of models, only the anhedonia measures and positive affect measure had significant or “trend-level” effects on erythema, such that increased anhedonia or lower positive affect was associated with increased erythema response (greater intercept). Specifically, Baseline SHAPS Anhedonia had trending (*p* = .074) and significant (*p* = .038) effects on raw and transformed erythema responses, respectively, while Baseline DARS Social Anhedonia had significant effects on both the raw and transformed erythema responses (*p* = .038 and *p* = .013, respectively). Baseline DARS Overall Anhedonia and Baseline PANAS Positive Affect had “trend-level” effects on raw and transformed erythema scores (*p*’s .055—.092). None of the other affect predictors showed significant or near-significant associations (all *p*’s > .10), suggesting the relationship between erythema and affect may be specific to the domain of positive emotion. Interestingly, in all multi-level models, NTTI Skin Spectrum showed—at most—only a “trend-level” effect on PI-MED erythema.

When each of the three baseline anhedonia measures was tested separately in multi-level models that included all other affect predictors (see Supplemental Tables 8-10), only Baseline DARS Social Anhedonia had a “trend-level” effect on the transformed erythema scores (*p* = .055), while no other affect predictors in the model had significant effects.

It should be noted that we chose to focus the main manuscript results on models estimating Composite Erythema (rather than multi-level models estimating the erythema effect across six exposure site measures), in order to collapse across any potential systematic measurement error between the odd and even numbered exposure sites (see Fig 2., Richey, et al., 2019).

|  | **Erythema (raw)** | | | **Erythema (transformed)** | | |
| --- | --- | --- | --- | --- | --- | --- |
| *Predictors* | *Estimates* | *CI* | *p* | *Estimates* | *CI* | *p* |
| (Intercept) | 0.54 | -0.54 – 1.61 | 0.329 | 0.50 | 0.40 – 0.60 | **<0.001** |
| ***Aperture** | -0.72 | -0.80 – -0.63 | **<0.001** | -0.03 | -0.04 – -0.03 | **<0.001** |
| NTTI Skin Spectrum | -0.05 | -0.11 – 0.01 | 0.087 | -0.00 | -0.01 – 0.00 | 0.125 |
| ***Age** | -0.06 | -0.11 – -0.01 | **0.012** | -0.01 | -0.01 – -0.00 | **0.036** |
| Sex | -0.19 | -1.27 – 0.88 | 0.722 | -0.02 | -0.12 – 0.08 | 0.708 |
| Minority | -0.11 | -2.17 – 1.95 | 0.916 | -0.01 | -0.20 – 0.18 | 0.905 |
| ***Baseline SHAPS Anhedonia** | 0.19 | -0.02 – 0.39 | 0.074 | 0.02 | 0.00 – 0.04 | **0.038** |
| **Random Effects** | | | | | | |
| σ^2^ | 2.03 | | | 0.02 | | |
| τ_00_ | 3.96 _id_ | | | 0.03 _id_ | | |
| ICC | 0.66 | | | 0.60 | | |
| N | 60 _id_ | | | 60 _id_ | | |
| Observations | 360 | | | 360 | | |
| Marginal R^2^ / Conditional R^2^ | 0.311 / 0.767 | | | 0.188 / 0.676 | | |

**Supplemental Table 1Multi-level models predicting raw and transformed erythema, with Baseline SHAPS Anhedonia added to the base model.**

|  | **Erythema (raw)** | | | **Erythema (transformed)** | | |
| --- | --- | --- | --- | --- | --- | --- |
| *Predictors* | *Estimates* | *CI* | *p* | *Estimates* | *CI* | *p* |
| (Intercept) | 0.65 | -0.40 – 1.70 | 0.227 | 0.52 | 0.42 – 0.61 | **<0.001** |
| ***Aperture** | -0.72 | -0.80 – -0.63 | **<0.001** | -0.03 | -0.04 – -0.03 | **<0.001** |
| NTTI Skin Spectrum | -0.04 | -0.10 – 0.02 | 0.165 | -0.00 | -0.01 – 0.00 | 0.262 |
| ***Age** | -0.07 | -0.12 – -0.02 | **0.004** | -0.01 | -0.01 – -0.00 | **0.014** |
| Sex | -0.11 | -1.19 – 0.97 | 0.842 | -0.01 | -0.11 – 0.09 | 0.826 |
| Minority | -0.54 | -2.51 – 1.42 | 0.588 | -0.06 | -0.24 – 0.12 | 0.523 |
| ~*Baseline DARS Overall Anhedonia | -0.05 | -0.11 – 0.00 | 0.068 | -0.01 | -0.01 – 0.00 | 0.055 |
| **Random Effects** | | | | | | |
| σ^2^ | 2.03 | | | 0.02 | | |
| τ_00_ | 3.95 _id_ | | | 0.03 _id_ | | |
| ICC | 0.66 | | | 0.60 | | |
| N | 60 _id_ | | | 60 _id_ | | |
| Observations | 360 | | | 360 | | |
| Marginal R^2^ / Conditional R^2^ | 0.312 / 0.767 | | | 0.182 / 0.676 | | |

**Supplemental Table 2 Multi-level models predicting raw and transformed erythema, with Baseline DARS Overall Anhedonia added to the base model.**

|  | **Erythema (raw)** | | | **Erythema (transformed)** | | |
| --- | --- | --- | --- | --- | --- | --- |
| *Predictors* | *Estimates* | *CI* | *p* | *Estimates* | *CI* | *p* |
| (Intercept) | 0.58 | -0.47 – 1.64 | 0.278 | 0.51 | 0.41 – 0.60 | **<0.001** |
| ***Aperture** | -0.72 | -0.80 – -0.63 | **<0.001** | -0.03 | -0.04 – -0.03 | **<0.001** |
| NTTI Skin Spectrum | -0.05 | -0.10 – 0.01 | 0.102 | -0.00 | -0.01 – 0.00 | 0.140 |
| ***Age** | -0.07 | -0.12 – -0.02 | **0.004** | -0.01 | -0.01 – -0.00 | **0.013** |
| Sex | -0.16 | -1.23 – 0.91 | 0.773 | -0.01 | -0.11 – 0.08 | 0.777 |
| Minority | -0.30 | -2.28 – 1.69 | 0.770 | -0.03 | -0.21 – 0.15 | 0.758 |
| ***Baseline DARS Social Anhedonia** | -0.18 | -0.35 – -0.00 | **0.049** | -0.02 | -0.04 – -0.00 | **0.013** |
| **Random Effects** | | | | | | |
| σ^2^ | 2.03 | | | 0.02 | | |
| τ_00_ | 3.91 _id_ | | | 0.03 _id_ | | |
| ICC | 0.66 | | | 0.59 | | |
| N | 60 _id_ | | | 60 _id_ | | |
| Observations | 360 | | | 360 | | |
| Marginal R^2^ / Conditional R^2^ | 0.316 / 0.767 | | | 0.203 / 0.675 | | |

**Supplemental Table 3 Multi-level models predicting raw and transformed erythema, with Baseline DARS Social Anhedonia added to the base model.**

|  | **Erythema (raw)** | | | **Erythema (transformed)** | | |
| --- | --- | --- | --- | --- | --- | --- |
| *Predictors* | *Estimates* | *CI* | *p* | *Estimates* | *CI* | *p* |
| (Intercept) | 0.63 | -0.42 – 1.68 | 0.241 | 0.52 | 0.42 – 0.62 | **<0.001** |
| ***Aperture** | -0.72 | -0.80 – -0.63 | **<0.001** | -0.03 | -0.04 – -0.03 | **<0.001** |
| NTTI Skin Spectrum | -0.05 | -0.11 – 0.01 | 0.080 | -0.00 | -0.01 – 0.00 | 0.149 |
| ***Age** | -0.07 | -0.12 – -0.02 | **0.005** | -0.01 | -0.01 – -0.00 | **0.017** |
| Sex | -0.28 | -1.35 – 0.79 | 0.612 | -0.03 | -0.13 – 0.07 | 0.592 |
| Minority | -0.25 | -2.26 – 1.77 | 0.810 | -0.04 | -0.23 – 0.15 | 0.713 |
| ~*Baseline PANAS Positive Affect | -0.07 | -0.14 – 0.01 | 0.070 | -0.01 | -0.01 – 0.00 | 0.092 |
| **Random Effects** | | | | | | |
| σ^2^ | 2.03 | | | 0.02 | | |
| τ_00_ | 3.96 _id_ | | | 0.03 _id_ | | |
| ICC | 0.66 | | | 0.61 | | |
| N | 60 _id_ | | | 60 _id_ | | |
| Observations | 360 | | | 360 | | |
| Marginal R^2^ / Conditional R^2^ | 0.312 / 0.767 | | | 0.175 / 0.676 | | |

**Supplemental Table 4 Multi-level models predicting raw and transformed erythema, with Baseline PANAS Positive Affect added to the base model.**

|  | **Erythema (raw)** | | | **Erythema (transformed)** | | |
| --- | --- | --- | --- | --- | --- | --- |
| *Predictors* | *Estimates* | *CI* | *p* | *Estimates* | *CI* | *p* |
| (Intercept) | 0.76 | -0.31 – 1.83 | 0.166 | 0.53 | 0.43 – 0.63 | **<0.001** |
| ***Aperture** | -0.72 | -0.80 – -0.63 | **<0.001** | -0.03 | -0.04 – -0.03 | **<0.001** |
| NTTI Skin Spectrum | -0.04 | -0.09 – 0.02 | 0.217 | -0.00 | -0.01 – 0.00 | 0.335 |
| ***Age** | -0.07 | -0.12 – -0.02 | **0.006** | -0.01 | -0.01 – -0.00 | **0.018** |
| Sex | -0.26 | -1.36 – 0.84 | 0.644 | -0.03 | -0.13 – 0.08 | 0.622 |
| Minority | -0.68 | -2.70 – 1.35 | 0.512 | -0.07 | -0.26 – 0.12 | 0.445 |
| Baseline PANAS Negative Affect | -0.01 | -0.09 – 0.07 | 0.826 | -0.00 | -0.01 – 0.01 | 0.917 |
| **Random Effects** | | | | | | |
| σ^2^ | 2.03 | | | 0.02 | | |
| τ_00_ | 4.21 _id_ | | | 0.04 _id_ | | |
| ICC | 0.68 | | | 0.62 | | |
| N | 60 _id_ | | | 60 _id_ | | |
| Observations | 360 | | | 360 | | |
| Marginal R^2^ / Conditional R^2^ | 0.284 / 0.768 | | | 0.148 / 0.677 | | |

**Supplemental Table 5 Multi-level models predicting raw and transformed erythema, with Baseline PANAS Negative Affect added to the base model.**

|  | **Erythema (raw)** | | | **Erythema (transformed)** | | |
| --- | --- | --- | --- | --- | --- | --- |
| *Predictors* | *Estimates* | *CI* | *p* | *Estimates* | *CI* | *p* |
| (Intercept) | 0.83 | -0.23 – 1.90 | 0.125 | 0.53 | 0.43 – 0.64 | **<0.001** |
| ***Aperture** | -0.72 | -0.80 – -0.63 | **<0.001** | -0.03 | -0.04 – -0.03 | **<0.001** |
| NTTI Skin Spectrum | -0.04 | -0.10 – 0.02 | 0.164 | -0.00 | -0.01 – 0.00 | 0.248 |
| ***Age** | -0.07 | -0.12 – -0.02 | **0.006** | -0.01 | -0.01 – -0.00 | **0.019** |
| Sex | -0.44 | -1.57 – 0.69 | 0.447 | -0.05 | -0.15 – 0.06 | 0.391 |
| Minority | -0.57 | -2.57 – 1.43 | 0.577 | -0.06 | -0.25 – 0.13 | 0.523 |
| Baseline Beck Depression Inventory II | 0.03 | -0.02 – 0.09 | 0.244 | 0.00 | -0.00 – 0.01 | 0.162 |
| **Random Effects** | | | | | | |
| σ^2^ | 2.03 | | | 0.02 | | |
| τ_00_ | 4.11 _id_ | | | 0.04 _id_ | | |
| ICC | 0.67 | | | 0.61 | | |
| N | 60 _id_ | | | 60 _id_ | | |
| Observations | 360 | | | 360 | | |
| Marginal R^2^ / Conditional R^2^ | 0.296 / 0.767 | | | 0.167 / 0.677 | | |

**Supplemental Table 6 Multi-level models predicting raw and transformed erythema, with Baseline Beck Depression Inventory II added to the base model.**

|  | **Erythema (raw)** | | | **Erythema (transformed)** | | |
| --- | --- | --- | --- | --- | --- | --- |
| *Predictors* | *Estimates* | *CI* | *p* | *Estimates* | *CI* | *p* |
| (Intercept) | 0.83 | -0.22 – 1.89 | 0.122 | 0.53 | 0.43 – 0.63 | **<0.001** |
| ***Aperture** | -0.72 | -0.80 – -0.63 | **<0.001** | -0.03 | -0.04 – -0.03 | **<0.001** |
| NTTI Skin Spectrum | -0.04 | -0.10 – 0.02 | 0.177 | -0.00 | -0.01 – 0.00 | 0.279 |
| ***Age** | -0.07 | -0.12 – -0.02 | **0.009** | -0.01 | -0.01 – -0.00 | **0.026** |
| Sex | -0.34 | -1.43 – 0.74 | 0.535 | -0.03 | -0.14 – 0.07 | 0.507 |
| Minority | -0.71 | -2.69 – 1.27 | 0.483 | -0.08 | -0.26 – 0.11 | 0.420 |
| Baseline Perceived Stress | 0.06 | -0.02 – 0.15 | 0.148 | 0.01 | -0.00 – 0.01 | 0.125 |
| **Random Effects** | | | | | | |
| σ^2^ | 2.03 | | | 0.02 | | |
| τ_00_ | 4.05 _id_ | | | 0.03 _id_ | | |
| ICC | 0.67 | | | 0.61 | | |
| N | 60 _id_ | | | 60 _id_ | | |
| Observations | 360 | | | 360 | | |
| Marginal R^2^ / Conditional R^2^ | 0.302 / 0.767 | | | 0.170 / 0.677 | | |

**Supplemental Table 7 Multi-level models predicting raw and transformed erythema, with Baseline Perceived Stress added to the base model.**

|  | **Erythema (raw)** | | | **Erythema (transformed)** | | |
| --- | --- | --- | --- | --- | --- | --- |
| *Predictors* | *Estimates* | *CI* | *p* | *Estimates* | *CI* | *p* |
| (Intercept) | 0.54 | -0.60 – 1.69 | 0.352 | 0.51 | 0.40 – 0.61 | **<0.001** |
| ***Aperture** | -0.72 | -0.80 – -0.63 | **<0.001** | -0.03 | -0.04 – -0.03 | **<0.001** |
| ~*NTTI Skin Spectrum | -0.06 | -0.12 – 0.00 | 0.067 | -0.00 | -0.01 – 0.00 | 0.112 |
| ***Age** | -0.06 | -0.11 – -0.01 | **0.015** | -0.00 | -0.01 – -0.00 | **0.045** |
| Sex | -0.33 | -1.51 – 0.85 | 0.581 | -0.04 | -0.15 – 0.07 | 0.513 |
| Minority | 0.08 | -2.10 – 2.25 | 0.946 | 0.00 | -0.20 – 0.20 | 0.998 |
| Baseline PANAS Positive Affect | -0.03 | -0.14 – 0.07 | 0.501 | -0.00 | -0.01 – 0.01 | 0.697 |
| Baseline PANAS Negative Affect | -0.05 | -0.15 – 0.05 | 0.368 | -0.00 | -0.01 – 0.00 | 0.335 |
| Baseline Perceived Stress | 0.04 | -0.10 – 0.17 | 0.593 | 0.00 | -0.01 – 0.02 | 0.568 |
| Baseline Beck Depression Inventory II | 0.01 | -0.07 – 0.09 | 0.838 | 0.00 | -0.01 – 0.01 | 0.667 |
| Baseline SHAPS Anhedonia | 0.14 | -0.09 – 0.37 | 0.241 | 0.02 | -0.01 – 0.04 | 0.156 |
| **Random Effects** | | | | | | |
| σ^2^ | 2.03 | | | 0.02 | | |
| τ_00_ | 4.08 _id_ | | | 0.03 _id_ | | |
| ICC | 0.67 | | | 0.61 | | |
| N | 60 _id_ | | | 60 _id_ | | |
| Observations | 360 | | | 360 | | |
| Marginal R^2^ / Conditional R^2^ | 0.321 / 0.775 | | | 0.201 / 0.688 | | |

**Supplemental Table 8 Multi-level models predicting raw and transformed erythema, with Baseline SHAPS Anhedonia added to a model with all other affective predictors.**

|  | **Erythema (raw)** | | | **Erythema (transformed)** | | |
| --- | --- | --- | --- | --- | --- | --- |
| *Predictors* | *Estimates* | *CI* | *p* | *Estimates* | *CI* | *p* |
| (Intercept) | 0.69 | -0.41 – 1.78 | 0.218 | 0.52 | 0.42 – 0.63 | **<0.001** |
| ***Aperture** | -0.72 | -0.80 – -0.63 | **<0.001** | -0.03 | -0.04 – -0.03 | **<0.001** |
| ~*NTTI Skin Spectrum | -0.05 | -0.11 – 0.01 | 0.133 | -0.00 | -0.01 – 0.00 | 0.235 |
| ***Age** | -0.07 | -0.12 – -0.02 | **0.009** | -0.01 | -0.01 – -0.00 | **0.028** |
| Sex | -0.31 | -1.48 – 0.86 | 0.608 | -0.04 | -0.14 – 0.07 | 0.527 |
| Minority | -0.35 | -2.42 – 1.73 | 0.743 | -0.05 | -0.24 – 0.15 | 0.636 |
| Baseline PANAS Positive Affect | -0.01 | -0.12 – 0.10 | 0.866 | 0.00 | -0.01 – 0.01 | 0.893 |
| Baseline PANAS Negative Affect | -0.06 | -0.16 – 0.04 | 0.258 | -0.01 | -0.02 – 0.00 | 0.222 |
| Baseline Perceived Stress | 0.07 | -0.06 – 0.20 | 0.305 | 0.01 | -0.01 – 0.02 | 0.261 |
| Baseline Beck Depression Inventory II | 0.01 | -0.07 – 0.09 | 0.767 | 0.00 | -0.01 – 0.01 | 0.574 |
| Baseline DARS Overall Anhedonia | -0.05 | -0.11 – 0.01 | 0.131 | -0.01 | -0.01 – 0.00 | 0.096 |
| **Random Effects** | | | | | | |
| σ^2^ | 2.03 | | | 0.02 | | |
| τ_00_ | 4.00 _id_ | | | 0.03 _id_ | | |
| ICC | 0.66 | | | 0.61 | | |
| N | 60 _id_ | | | 60 _id_ | | |
| Observations | 360 | | | 360 | | |
| Marginal R^2^ / Conditional R^2^ | 0.329 / 0.775 | | | 0.208 / 0.688 | | |

**Supplemental Table 9 Multi-level models predicting raw and transformed erythema, with Baseline DARS Overall Anhedonia added to a model with all other affective predictors.**

|  | **Erythema (raw)** | | | **Erythema (transformed)** | | |
| --- | --- | --- | --- | --- | --- | --- |
| *Predictors* | *Estimates* | *CI* | *p* | *Estimates* | *CI* | *p* |
| (Intercept) | 0.60 | -0.51 – 1.71 | 0.286 | 0.51 | 0.41 – 0.62 | **<0.001** |
| ***Aperture** | -0.72 | -0.80 – -0.63 | **<0.001** | -0.03 | -0.04 – -0.03 | **<0.001** |
| ~*NTTI Skin Spectrum | -0.05 | -0.11 – 0.01 | 0.079 | -0.00 | -0.01 – 0.00 | 0.128 |
| ***Age** | -0.07 | -0.12 – -0.02 | **0.008** | -0.01 | -0.01 – -0.00 | **0.024** |
| Sex | -0.30 | -1.48 – 0.87 | 0.615 | -0.03 | -0.14 – 0.08 | 0.570 |
| Minority | -0.12 | -2.21 – 1.97 | 0.909 | -0.02 | -0.21 – 0.17 | 0.839 |
| Baseline PANAS Positive Affect | -0.03 | -0.13 – 0.07 | 0.599 | -0.00 | -0.01 – 0.01 | 0.854 |
| Baseline PANAS Negative Affect | -0.04 | -0.14 – 0.06 | 0.478 | -0.00 | -0.01 – 0.01 | 0.477 |
| Baseline Perceived Stress | 0.05 | -0.08 – 0.18 | 0.453 | 0.01 | -0.01 – 0.02 | 0.393 |
| Baseline Beck Depression Inventory II | 0.01 | -0.07 – 0.09 | 0.884 | 0.00 | -0.01 – 0.01 | 0.747 |
| ~*Baseline DARS Social Anhedonia | -0.14 | -0.33 – 0.05 | 0.158 | -0.02 | -0.03 – 0.00 | 0.055 |
| **Random Effects** | | | | | | |
| σ^2^ | 2.03 | | | 0.02 | | |
| τ_00_ | 4.03 _id_ | | | 0.03 _id_ | | |
| ICC | 0.67 | | | 0.60 | | |
| N | 60 _id_ | | | 60 _id_ | | |
| Observations | 360 | | | 360 | | |
| Marginal R^2^ / Conditional R^2^ | 0.327 / 0.775 | | | 0.216 / 0.687 | | |

**Supplemental Table 10 Multi-level models predicting raw and transformed erythema, with Baseline DARS Social Anhedonia added to a model with all other affective predictors.**

***Prospective Associations between Anhedonia and PI-MED Erythema.*** Models examining prospective prediction of End-Point Composite Erythema by Baseline Composite Erythema and other baseline predictors including demographics, NTTI Skin Spectrum, and Mindfulness Intervention Condition (as a binary predictor) and excluding BMI are presented in Supplemental Table 11 (and the same set of models including BMI are presented in Supplemental Table 12). The Final Base Model including all covariates (except BMI) was significant (*p* <.001) and accounted for 57% of the variance, with only Baseline Composite Erythema as a significant predictor of End-Point Composite Erythema (β = .69, *p* < .001). The model that added DARS Overall Anhedonia (*p*= .77) and the model that added DARS Social Anhedonia (﻿*p*= .41) were not significant improvements upon the Final Base Model. The model that added SHAPS Anhedonia (﻿*p*= .04) was a significant improvement upon the Final Base Model and accounted for an additional 5% of the variance (R2= .62), with Baseline SHAPS Anhedonia as a significant *negative* predictor of End-Point Composite Erythema (β = -.25, *p* = .04). Thus, in contrast to the *positive* concurrent association between Baseline SHAPS Anhedonia and Baseline Composite Erythema, for the prospective associations, greater Baseline SHAPS Anhedonia predicted *lower* End-Point Composite Erythema.

***Discussion of*** ***Prospective Associations between Anhedonia and PI-MED Erythema.*** In prospective associations, only baseline SHAPS Anhedonia (but not other anhedonia measures) significantly improved prediction of PI-MED erythema at the study end-point, over and above a base model that included baseline PI-MED erythema, NTTI Skin Spectrum score, and other non-affective predictors. However, the direction of anhedonia’s prospective association was opposite to that of its concurrent association, with greater baseline SHAPS Anhedonia predicting *lower* end-point erythema. This reversed association may reflect regression to the mean whereby individuals with higher anhedonia and associated higher erythema at baseline had, by the end of the study, seen their high levels of erythema subside to lower levels, producing a negative association between baseline anhedonia and end-point erythema.

**Supplemental Table 11 Prospective associations between anhedonia measures and PI-MED erythema (excluding BMI)**

|  |  |  |  |  | |  |  |  |  |  |  |  |  |
| --- | --- | --- | --- | --- | --- | --- | --- | --- | --- | --- | --- | --- | --- |
|  |  |  |  |  | |  |  |  |  |  |  |  |  |
|  |  |  |  |  | |  | β Coef | B Coef | SE | *t* | *p* |  | 95% CI |
| ****Final Base Model (without BMI covariate) (n = 42)** | | | | **F(6,35)= 7.63, *p <* .001, R2= .57, Adj-R2=.49, SE of Estimate= .16** | | | | | | | | | |
|  |  | ***Constant** | |  |  | |  | .33 | .16 | 2.10 | .04 |  | [.01, .66] |
|  |  | ****Baseline Composite Erythema** | |  |  | | .67 | .62 | .11 | 5.47 | < .001 |  | [.39, .85] |
|  |  | NTTI Skin Spectrum | |  |  | | -.27 | .00 | .00 | -1.30 | .20 |  | [-.01, .00] |
|  |  | Sex | |  |  | | -.05 | -.02 | .05 | -.42 | .68 |  | [-.13, .09] |
|  |  | Age | |  |  | | -.03 | .00 | .00 | -.22 | .83 |  | [-.01, .00] |
|  |  | Minority | |  |  | | .05 | .02 | .11 | .23 | .82 |  | [-.19, .24] |
|  |  | Mindfulness Intervention Condition | |  |  | | .09 | .04 | .05 | .74 | .46 |  | [-.07, .14] |
| ***Hierarchical Model: Final Base + SHAPS (n = 42)** | | | | **﻿ΔF(1,34)= 4.63, *p*= .04, R2= .62, Adj-R2=.54, ΔR2= .05, SE of Estimate= .16** | | | | | | | | | |
|  |  | ***Constant** | |  |  | |  | .36 | .15 | 2.37 | .02 |  | [.05, .67] |
|  |  | ****Baseline Composite Erythema** | |  |  | | .73 | .67 | .11 | 6.07 | < .001 |  | [.45, .90] |
|  |  | NTTI Skin Spectrum | |  |  | | -.18 | .00 | .00 | -.88 | .38 |  | [-.01, .00] |
|  |  | Sex | |  |  | | -.07 | -.03 | .05 | -.63 | .53 |  | [-.14, .07] |
|  |  | Age | |  |  | | -.07 | .00 | .00 | -.56 | .58 |  | [-.01, .00] |
|  |  | Minority | |  |  | | -.04 | -.02 | .10 | -.20 | .85 |  | [-.23, .19] |
|  |  | Mindfulness Intervention Condition | |  |  | | .06 | .03 | .05 | .58 | .57 |  | [-.07, .13] |
|  |  | ***Baseline SHAPS Anhedonia** |  |  |  | | -.25 | -.02 | .01 | -2.15 | .04 |  | [-.05, -.00] |
| **Hierarchical Model: Final Base + DARS Overall (n = 42)** | | | | **﻿ΔF(1,34)= .09, *p*= .77, R2= .57, Adj-R2=.48, ΔR2= .00, SE of Estimate= .17** | | | | | | | | | |
|  |  | ***Constant** | |  |  | |  | .40 | .27 | 1.47 | .15 |  | [-.15, .95] |
|  |  | ****Baseline Composite Erythema** | |  |  | | .66 | .61 | .12 | 5.11 | < .001 |  | [.37, .85] |
|  |  | NTTI Skin Spectrum | |  |  | | -.28 | .00 | .00 | -1.30 | .20 |  | [-.01, .00] |
|  |  | Sex | |  |  | | -.05 | -.02 | .05 | -.40 | .69 |  | [-.13, .09] |
|  |  | Age | |  |  | | -.03 | .00 | .00 | -.25 | .81 |  | [-.01, .00] |
|  |  | Minority | |  |  | | .05 | .02 | .11 | .21 | .84 |  | [-.19, .24] |
|  |  | Mindfulness Intervention Condition | |  |  | | .09 | .04 | .05 | .74 | .46 |  | [-.07, .15] |
|  |  | Baseline DARS Overall Anhedonia |  |  |  | | -.04 | .00 | .00 | -.30 | .77 |  | [-.01, .00] |
| **Hierarchical Model: Final Base + DARS Social (n = 42)** | | | | **﻿ΔF(1,34)= .68, *p*= .41, R2= .58, Adj-R2=.49, ΔR2= .01, SE of Estimate= .16** | | | | | | | | | |
|  |  | Constant | |  |  | |  | .21 | .22 | .99 | .33 |  | [-.22, .65] |
|  |  | ****Baseline Composite Erythema** | |  |  | | .71 | .66 | .12 | 5.39 | < .001 |  | [.41, .90] |
|  |  | NTTI Skin Spectrum | |  |  | | -.24 | .00 | .00 | -1.12 | .27 |  | [-.01, .00] |
|  |  | Sex | |  |  | | -.05 | -.02 | .05 | -.43 | .67 |  | [-.13, .09] |
|  |  | Age | |  |  | | -.01 | .00 | .00 | -.07 | .94 |  | [-.01, .00] |
|  |  | Minority | |  |  | | .04 | .02 | .11 | .19 | .85 |  | [-.20, .23] |
|  |  | Mindfulness Intervention Condition | |  |  | | .08 | .04 | .05 | .73 | .47 |  | [-.07, .14] |
|  |  | Baseline DARS Social Anhedonia |  |  |  | | .10 | .01 | .01 | .83 | .41 |  | [-.01, .03] |
|  |  |  |  |  |  | |  |  |  |  |  |  |  |
| ** *p* < .01 | | | |  | |  |  |  |  |  |  |  |  |
| * *p* < .05 | | | |  | |  |  |  |  |  |  |  |  |

**Supplemental Table 12 Prospective associations between anhedonia measures and PI-MED erythema (including BMI)**

|  |  |  |  |  |  | | | β Coef | B Coef | | SE | t | p |  | 95% CI |  |
| --- | --- | --- | --- | --- | --- | --- | --- | --- | --- | --- | --- | --- | --- | --- | --- | --- |
| ****Final Base Model (with BMI covariate) (n = 35)** | | | | | **F(7,27)= 8.95, p < .001, R2= .70, Adj-R2=.62, SE of Estimate= .15** | | | | | | | | | | |  |
|  |  | *Constant | |  |  | | |  | | .51 | .17 | 3.01 | .01 |  | [.16, .85] |  |
|  |  | **Baseline Composite Erythema | |  |  | | | .66 | | .61 | .11 | 5.69 | < .001 |  | [.39, .83] |  |
|  |  | NTTI Skin Spectrum | |  |  | | | -.16 | | .00 | .00 | -.72 | .47 |  | [-.01, .00] |  |
|  |  | Sex | |  |  | | | -.07 | | -.04 | .05 | -.66 | .51 |  | [-.15, .08] |  |
|  |  | Age | |  |  | | | -.05 | | .00 | .00 | -.39 | .70 |  | [-.01, .00] |  |
|  |  | Minority | |  |  | | | -.15 | | -.07 | .11 | -.66 | .51 |  | [-.30, .15] |  |
|  |  | Body Mass Index | |  |  | | | -.18 | | .00 | .00 | -1.57 | .13 |  | [-.01, .00] |  |
|  |  | Mindfulness Intervention Condition | |  |  | | | -.03 | | -.01 | .05 | -.27 | .79 |  | [-.12, .09] |  |
| ***Hierarchical Model: Final Base + SHAPS (n = 35)** | | | | | **﻿ΔF(1,26)= 4.40, p= .05, R2= .74, Adj-R2=.66, ΔR2= .04, SE of Estimate= .14** | | | | | | | | | | |  |
|  |  | *Constant | |  |  | | |  | | .48 | .16 | 3.02 | .01 |  | [.15, .81] |  |
|  |  | **Baseline Composite Erythema | |  |  | | | .72 | | .67 | .10 | 6.38 | < .001 |  | [.45, .88] |  |
|  |  | NTTI Skin Spectrum | |  |  | | | -.08 | | .00 | .00 | -.39 | .70 |  | [-.01, .00] |  |
|  |  | Sex | |  |  | | | -.12 | | -.06 | .05 | -1.14 | .27 |  | [-.17, .05] |  |
|  |  | Age | |  |  | | | -.06 | | .00 | .00 | -.53 | .60 |  | [-.01, .00] |  |
|  |  | Minority | |  |  | | | -.21 | | -.11 | .11 | -1.01 | .32 |  | [-.33, .11] |  |
|  |  | Body Mass Index | |  |  | | | -.11 | | .00 | .00 | -.94 | .36 |  | [-.01, .00] |  |
|  |  | Mindfulness Intervention Condition | |  |  | | | -.05 | | -.02 | .05 | -.46 | .65 |  | [-.13, .08] |  |
|  |  | *Baseline SHAPS Anhedonia |  |  |  | | | -.24 | | -.02 | .01 | -2.10 | .05 |  | [-.05, -.00] |  |
| **Hierarchical Model: Final Base + DARS Overall (n = 35)** | | | | | | | **﻿ΔF(1,26)= .34, p= .57, R2= .70, Adj-R2=.61, ΔR2= .00, SE of Estimate= .15** | | | | | | | | | |
|  |  | *Constant | |  |  | | |  | | .64 | .28 | 2.25 | .03 |  | [.06, 1.22] |  |
|  |  | **Baseline Composite Erythema | |  |  | | | .64 | | .59 | .11 | 5.19 | < .001 |  | [.36, .83] |  |
|  |  | NTTI Skin Spectrum | |  |  | | | -.18 | | .00 | .00 | -.81 | .43 |  | [-.01, .00] |  |
|  |  | Sex | |  |  | | | -.06 | | -.03 | .06 | -.55 | .59 |  | [-.14, .08] |  |
|  |  | Age | |  |  | | | -.05 | | .00 | .00 | -.41 | .68 |  | [-.01, .00] |  |
|  |  | Minority | |  |  | | | -.13 | | -.07 | .11 | -.59 | .56 |  | [-.30, .17] |  |
|  |  | Body Mass Index | |  |  | | | -.17 | | .00 | .00 | -1.51 | .14 |  | [-.01, .00] |  |
|  |  | Mindfulness Intervention Condition | |  |  | | | -.02 | | -.01 | .05 | -.18 | .86 |  | [-.12, .10] |  |
|  |  | Baseline DARS Overall Anhedonia |  |  |  | | | -.07 | | .00 | .00 | -.58 | .57 |  | [-.01, .00] |  |
| **Hierarchical Model: Final Base + DARS Social (n = 35)** | | | | | | **﻿ΔF(1,26)= .46, p= .50, R2= .70, Adj-R2=.61, ΔR2= .00, SE of Estimate= .15** | | | | | | | | | |  |
|  |  | Constant | |  |  | | |  | | .39 | .24 | 1.65 | .11 |  | [-.10, .88] |  |
|  |  | **Baseline Composite Erythema | |  |  | | | .70 | | .64 | .12 | 5.42 | < .001 |  | [.40, .89] |  |
|  |  | NTTI Skin Spectrum | |  |  | | | -.12 | | .00 | .00 | -.51 | .61 |  | [-.01, .00] |  |
|  |  | Sex | |  |  | | | -.09 | | -.04 | .06 | -.77 | .45 |  | [-.16, .07] |  |
|  |  | Age | |  |  | | | -.03 | | .00 | .00 | -.25 | .80 |  | [-.01, .00] |  |
|  |  | Minority | |  |  | | | -.17 | | -.08 | .11 | -.75 | .46 |  | [-.32, .15] |  |
|  |  | Body Mass Index | |  |  | | | -.17 | | .00 | .00 | -1.44 | .16 |  | [-.01, .00] |  |
|  |  | Mindfulness Intervention Condition | |  |  | | | -.03 | | -.01 | .05 | -.28 | .78 |  | [-.13, .10] |  |
|  |  | Baseline DARS Social Anhedonia |  |  |  | | | .09 | | .01 | .01 | .68 | .50 |  | [-.01, .03] |  |
|  |  |  |  |  |  | | |  | |  |  |  |  |  |  |  |
| ** p < .01 | | | |  |  | | |  | |  |  |  |  |  |  |  |
| * p ≤ .05 | | | |  |  | | |  | |  |  |  |  |  |  |  |
